# Theoretical and computational validation of the Kuhn barrier friction mechanism in unfolded proteins

**DOI:** 10.1038/s41598-017-00287-5

**Published:** 2017-03-21

**Authors:** Stanislav M. Avdoshenko, Atanu Das, Rohit Satija, Garegin A. Papoian, Dmitrii E. Makarov

**Affiliations:** 1 Institute for Computational Engineering and Sciences, University of Texas at Austin, Austin, Texas 78712 USA; 2 Department of Chemistry, University of Texas at Austin, Austin, Texas 78712 USA; 3 Department of Chemistry and Biochemistry, University of Maryland, College Park, MD, 20742 USA; 4 Leibniz Institute for Solid State and Materials Research (IFW Dresden), Helmholtzstraße 20, 01069 Dresden, Germany

## Abstract

A long time ago, Kuhn predicted that long polymers should approach a limit where their global motion is controlled by solvent friction alone, with ruggedness of their energy landscapes having no consequences for their dynamics. In contrast, internal friction effects are important for polymers of modest length. Internal friction in proteins, in particular, affects how fast they fold or find their binding targets and, as such, has attracted much recent attention. Here we explore the molecular origins of internal friction in unfolded proteins using atomistic simulations, coarse-grained models and analytic theory. We show that the characteristic internal friction timescale is directly proportional to the timescale of hindered dihedral rotations within the polypeptide chain, with a proportionality coefficient *b* that is independent of the chain length. Such chain length independence of *b* provides experimentally testable evidence that internal friction arises from concerted, crankshaft-like dihedral rearrangements. In accord with phenomenological models of internal friction, we find the global reconfiguration timescale of a polypeptide to be the sum of solvent friction and internal friction timescales. At the same time, the time evolution of inter-monomer distances within polypeptides deviates both from the predictions of those models and from a simple, one-dimensional diffusion model.

## Introduction

Protein folding may be justifiably viewed as a finite-size first-order phase transition, with folding kinetics following a classic nucleation mechanism^1, 2^. This view, however, masks the enormous complexity of folding dynamics at the molecular scale, where an unfolded protein chain samples a very large number of conformations before reaching the native state. Yet this number of conformations actually pales compared to the astronomically large number of conformations that the unfolded chain can access in principle. Levinthal considered this paradox of exhaustive search taking longer than the age of the universe, proposing that some combination of non-equilibrium dynamics and locally favorable interactions lead to biologically accessible folding times^3^. Subsequently, Anfinsen and Go pursued a purely thermodynamics view, emphasizing the congruency between stabilizing local and tertiary interactions, resulting in cooperative transition from the unfolded phase to the folded state^4, 5^. A modern statistical mechanical theory of protein folding has built upon these insights, suggesting that, in general, energy landscapes of globular proteins are globally correlated, where not only the native state is unusually low in energy compared to random conformations of the protein chain, but also conformations that partially resemble the native structure are also rather low in energy^6, 7^. This funnel-like organization of globular proteins’ conformational substates is extremely unlikely for random protein sequences, being a result of sequence evolution over billions of years^6^. The energy landscape theory of protein folding raises a new issue of local energetic ruggedness, where deep energetic traps might kinetically arrest the folding process^8^. Hence, a quantitative criterion of the ratio of the funnel depth to the magnitude of local energetic ruggedness determines whether a particular protein sequence will be foldable at laboratory or biological timescales, with an important related energy scale controlling the coil-to-globule collapse temperature^7, 9^.

The funnel theory is mesoscopic, leaving room for various alternative polymer theories of microscopic chain dynamics. Among interesting questions are whether the unfolded state is in a swollen (coiled) or molten globular state, and in the former case, whether folding is preceded by globular collapse or takes place concomitantly with it. Hence, both analytical and numerical models rooted in polymer theory can provide important insights into the nature of dynamics in the unfolded ensemble and into the specific collective processes driving the folding reaction. These approaches are particularly fruitful in addressing kinetic questions, where many prior studies of protein folding assumed either one-dimensional diffusion along some reaction coordinate^8^ (which is postulated, equated to an experimental signal, or computed based on an optimality criterion^10^) or relied on discrete kinetic networks^11–14^, which are often deduced from all-atom simulations. One-dimensional models are attractive because of their direct connection to experimental data, but, because they lump all the complexity of protein dynamics into a few empirical parameters (such as the effective diffusion coefficient), they often lack molecular insight. Moreover, the assumption of one-dimensional diffusion is not always justified^15–18^. Discrete state models, on the contrary, often involve too much molecular and kinetic information to offer adequate insight.

Polymer theory models offer an attractive middle ground between all atom descriptions and low-dimensional models. Such models highlight certain universal properties of chain molecules that are independent of their precise chemical identity. Of course, therein lies their weakness: they ignore sequence effects or specific intrachain interactions; yet despite this drawback they are often remarkably successful in accounting for many structural and dynamical features of proteins. For example, despite sequence diversity, the radius of gyration of most chemically denatured proteins was found^19^ to scale with the chain length *N* as *N* ^0.6^, in accord with Flory’s scaling law for random coil^20^ (although this scaling may not be applicable to proteins under physiological conditions^21, 22^). Likewise, we showed that the experimental observations of the dynamics of loop formation within both unfolded proteins and single-stranded DNA obey simple, universal relationships derived from polymer theory^23^.

Polymer theory makes simple, experimentally testable predictions regarding protein dynamics in the unfolded state. In particular, the global relaxation time *τ*_*r*_ (or *reconfiguration time*, in the language adopted by the protein folding community) of a sufficiently long unstructured polymer chain in solution is predicted to be comparable to the time it takes the chain to diffuse over a length equal to its own size^24^:

1 $${\tau }_{r}\simeq {\tau }_{RZ}=c\frac{\langle{R}^{2}\rangle}{{D}_{tr}}$$

Here we can take the mean-square end-to-end distance, $\langle{R}^{2}\rangle$, as a measure of the size, *D*_*tr*_ is the translational diffusion coefficient, and *c* is a numerical factor that depends on the specific model and on the precise definition of the reconfiguration time. If, for example, *τ*_*r*_ is identified with the timescale associated with the slowest relaxation mode in the Rouse model, then one finds^25^ *c* =* π* ^−2^/3. If, as in some studies, *τ*_*r*_ is defined as the end-to-end vector relaxation time^26^, or as the end-to-end distance relaxation time, the resulting values of *c* differ from the above one by only a numerical factor of order one (see ref. 17 and the Results section). We will refer to the limit where equation (1) is satisfied as the Rouse-Zimm (RZ) regime, as it is the regime where the standard Rouse or Zimm theories of chain dynamics apply^25^. Equation (1) provides a reasonable order-of-magnitude estimate of the reconfiguration time of chemically denatured proteins, but it manifestly underestimates the reconfiguration times of some of the intrinsically disordered proteins or proteins that are unfolded near native conditions^27^, presumably because of intra-chain interactions or some other new effects.

The remarkable feature of equation (1) is its insensitivity to any microscopic details of protein dynamics – a result known as the Kuhn theorem^24^. Indeed, assuming that *D*_*tr*_ depends only on the solvent temperature, viscosity, and the protein radius of gyration (as would be implied by the Stokes-Einstein formula), these parameters also completely determine the reconfiguration time, a characteristic of a protein’s *internal* dynamics.

The Kuhn theorem is true under the assumption of sufficiently long chains, which, of course, does not necessarily apply to finite-length polypeptides. It is then instructive to consider the opposite limit of a very short peptide, say a di-peptide. In this case, significantly different configurations of the molecule result from its *φ−* and *ψ*− dihedral angles occupying distinct regions of the Ramachandran plot. The characteristic time over which such a molecule significantly changes its conformation (as quantified, for example, by the fluctuation timescale of its end-to-end distance) is then controlled by the typical time *τ*_*dih*_ it takes the molecule to jump into a different Ramachandran plot region. Such jumps usually involve activated barrier crossing. In contrast, internal barriers do not affect the time of equation (1) (except through the relatively weak dependence of the average chain dimensions on the precise shape of the dihedral energy landscape). Following de Gennes^24^, we will refer to the short chain limit, where the chain dynamics timescale is dominated by *τ*_*dih*_, as the *barrier friction* limit. For notational brevity, we will reserve the term “barrier friction” to refer to this specific mechanism that involves overcoming dihedral barriers, keeping, however, in mind that many other intra-chain interactions may also give rise to microscopic kinetic barriers.

Barrier friction is related to (but not necessarily identical with) the notion of *internal friction* in proteins. The most common experimental definition of internal friction is as a viscosity-independent component of the friction; that is, if the dependence of *τ*_*r*_ on the solvent viscosity *η* is of the form *τ*_*r*_ = *τ*_*i*_ + *aη*, then *τ*_*i*_ is the internal friction timescale^28, 29^. Since dihedral rotations within a solvated polypeptide chain are, in general, mediated by the hydrodynamic drag on its various parts, there is no *a priori* reason to expect that *τ*_*i*_ would be entirely controlled by dihedral rotations. Nevertheless, recent computational evidence suggests that (i) dihedral relaxation times are weakly dependent on solvent viscosity^30, 31^ and (ii) the height of the dihedral barriers controls the internal friction timescale^30^. These observations may at least partially explain why the two different definitions of friction, internal friction (operationally defined as solvent viscosity independent component of reconfiguration time) and barrier friction (stemming from hindered dihedral rotations) may coincide^27^. Experimental studies that probed not only the slowest relaxation time but also the entire spectrum of relaxation times^27, 32^ further suggest that the dynamics of unfolded proteins can be interpreted in terms of the Rouse or Zimm models with internal friction^18, 24, 33, 34^ (RIF and ZIF), whose physical foundation is the Kuhn picture of barrier friction^35^. RIF and ZIF provide a semi-phenomenological description of internal friction effects and build on the classic Rouse and Zimm models of polymer dynamics.

Both Rouse and Zimm models are coarse-grained descriptions of polymers, which are represented as connected beads subjected to Brownian motion in solution. In addition to the solvent-induced forces, RIF and ZIF introduce an internal friction force, which resists deformation of inter-bead bonds. The physical mechanism of this force is that described by Kuhn: stretching a chain segment requires rearrangement of one or several dihedral angles within this segment, which is accomplished via activated barrier crossing. Bazua and Williams predicted a RIF/ZIF-type internal friction force using a rotational-isomeric-state model^35^, but even simpler arguments^24^ predict that the internal friction force on a chain segment must be inversely proportional to its length, *l*. Specifically, imagine that a stretching force *F* is applied to the ends of a segment, causing dihedral rotations that preferentially lead to a more extended segment state. The average velocity *u* at which the ends will be moving apart must be proportional to the number of dihedral transitions per unit time, which is proportional to the segment length *l*. In the linear response regime, it is also proportional to the force, so we have *u* ∝ *lF* or *F* ∝ *u*/*l*, resulting in a friction coefficient inversely proportional to *l*. Now, the relaxation time of the segment can be estimated as this friction coefficient divided by the segment’s effective spring constant. Since this spring constant is, likewise, inversely proportional to *l* ^24^ (assuming a chain with Gaussian statistics), this results in a single timescale that is independent of chain length: this is the internal friction timescale *τ*_*i*_; if, as suggested by simulations, this timescale is itself viscosity independent, it should coincide with the zero-viscosity intercept of *τ*_*r*_(*η*).

Despite these developments there is currently little consensus about what exactly internal friction is and how it is related to microscopic chain dynamics. Specifically, the following questions remain open:

1. The microscopic mechanisms of internal friction in unfolded proteins (and even in simpler polymeric systems^36^) remain elusive. In particular, while the idea that dihedral rotations lead to internal friction effects is not new, no quantitative connection between the microscopic parameters describing the dynamics of the dihedrals and the experimental measures of the internal friction (such as the internal friction time *τ*_*i*_ within the RIF/ZIF picture) is known. It is tempting to equate *τ*_*i*_ with the dihedral hopping time *τ*_*dih*_, but little experimental or theoretical evidence exists in support of this idea – just because the two timescales are related does not mean they are the same! In fact, in a series of papers by Allegra and collaborators^37–39^, an entirely different model was proposed to explain polymer relaxation in terms of dihedral dynamics; this model is at odds both with the relaxation spectrum predicted by RIF or ZIF and with the assertion that internal friction can be described in terms of a single, chain length independent timescale. Likewise, other models of polymer dynamics^24, 36, 40, 41^ postulate alternative internal friction mechanisms and do not necessarily lead to a single internal friction timescale.
2. A related question is concerned with the inherent limitations of the RIF/ZIF models. Despite their success in fitting experimental data, these models have a fundamental flaw: they fail to describe the rotational dynamics of a polymer chain and predict that, in the limit of high internal friction, the chain rotation timescale would coincide with *τ*_*i*_. This is obviously not true: even if the chain configuration is frozen with all dihedrals angles fixed, it can still rotate, with solvent friction determining the rotational timescale. It was thus argued^42^ that RIF and ZIF should be viewed as one-dimensional models that are only applicable to the internal chain dynamics. But since the concept of a dihedral angle is meaningless in a one-dimensional space, the connection between dihedral dynamics and the ZIF or RIF parameters becomes even more vague.
3. Most models of internal friction force postulate, without justification, additivity of internal and solvent friction. Yet an alternative common view of internal friction based on diffusion on rough landscapes^43^ postulates multiplicativity of the two effects! Without the additivity assumption, no theory exists that simultaneously includes both internal and solvent friction and interpolates between the solvent friction dominated and internal friction dominated limits; How, then, does the reconfiguration time *τ*_*r*_ depend on chain parameters in the (arguably most relevant experimentally) intermediate case between these two limits?
4. While the Rouse or Zimm models provide a reasonable view of polymer dynamics in the absence of internal friction effects, a mechanistic description of the chain dynamics in the internal-friction-dominated regime is lacking. In particular, although it is reasonable to view chain reconfiguration in this limit as resulting from dihedral rotations, whether such rotations must occur in a concerted fashion or can be independent of one another is an unsettled issue. Independent dihedral rotations require large motions of the entire polymer chain and, therefore, they must entail high solvent friction, but concerted rotations, while reducing the friction, involve higher activation energies^44^. Simulations indicated that dihedral rotations are concerted, involve simultaneous dihedral hops, and lead to localized, crankshaft-like movements of the chain^30^; however, whether such correlations are essential to explaining internal friction remains an open issue – if all the dihedrals changed independently, would that also lead to internal friction? Are there any experimentally measurable consequences of correlated dihedral motions?

In this paper, our aim is to address these questions, first for simpler, peptide-like model homopolymers, and then to extend the analysis to atomistic models. Our simulations show additivity of the Kuhn-type barrier friction and the Rouse/Zimm friction effects; moreover, when the barrier friction component of the protein reconfiguration time is equated with the RIF/ZIF internal friction time *τ*_*i*_, it is found to coincide, within a numerical, chain length independent factor, with the dihedral hopping time *τ*_*dih*_. This establishes a relationship between a phenomenological internal friction timescale postulated by RIF or ZIF and the microscopic chain dynamics. We further show that this relationship is only possible if dihedral angles change in a correlated fashion. Given the earlier findings that the dihedral relaxation timescale only shows weak viscosity dependence^30, 31^, our results further reconcile the experimental definition of internal friction as the zero-viscosity intercept of the reconfiguration time with the concept of Kuhn-type barrier friction. Finally, although the ZIF and RIF models correctly reproduce many qualitative features of reconfiguration dynamics, our results suggest that their utility for the analysis of experimental data has limitations. In particular, in contrast to the prediction of diffusive end-to-end distance dynamics in the high internal friction limit, all of the peptides studied here show subdiffusive dynamics.

## Methods

Three types of simulations were used in this work: atomistic simulations of short polypeptides, Langevin dynamics simulations of coarse-grained, C_α_-only peptide models, and kinetic Monte Carlo simulations of a rotational isomeric state models (RISM).

### Atomistic simulations

Molecular dynamics simulations were performed using the GROMACS software package, version 4.5.5^45^, using the Amber03 parameter set^46^ and an extended simple point charge (SPC/E) explicit water model. Starting from the NMR structure of the 66-residue Thermotoga maritima CSP (pdb access code 1G6P), the initial peptide models were built by cutting the protein into six equal 11-residue peptide fragments. Since studying shorter peptide fragments amplifies sequence effects (which may otherwise be averaged out in longer polypeptides), we have also performed simulations of an 11-residue peptide fragment with the Gly-Ser repeat, which is often viewed as a model polypeptide with properties close to those of a random coil^42, 47^. Finally, to assess the role of dihedral rotations on the reconfiguration timescale, we have studied a Gly-Ser repeat of the same length but with a reduced dihedral-barrier. After initial minimization and equilibration, production runs of 2 *μs* were performed, as in an earlier study^30^, at *T* = 300 K and *P* = 0.138 atm using the modified Berendsen thermostat^48^ and the Parrinello-Rahman barostat^49^. Further details are described in the Supplementary Information (SI).

### Coarse-grained model

Our random-coil, C_α_-only homopeptide model is similar to those described earlier^26, 50–52^ and represents each amino acid residue as a single bead; the model generally employs a 3-letter alphabet for the amino acid sequence, consisting of hydrophobic, neutral, and polar beads; however, to describe the unfolded polypeptide, sequences of various lengths consisting entirely of neutral beads were used. The potential energy of the chain included a harmonic spring potential describing the chain connectivity, a harmonic bending potential imposing the constraints inherent to peptide geometry, and a repulsive *r* ^−12^ potential that accounts for the excluded volume interactions. As in the original studies where this type of model was introduced^52^, the dependence of the energy on the dihedral angles *φ* is described by a potential of the form *V*_*dih*_ = *ε*(1 − cos3*φ*)/2, where the dihedral barrier height *ε* was varied to study how the dynamics of hindered rotations affects the peptide’s global relaxation timescale. The chain dynamics was governed by a Langevin equation. See the SI for further details.

### RISM simulations

In the (alpha-carbon only, coarse-grained) rotational isomeric state model (RISM), the configuration of the polypeptide chain is entirely specified by its dihedral angles, {*φ*_1_, *φ*_2, …_, *φ*_*N*−2_}, where *N* is the number of monomers. Same geometry (i.e. same bending angles) was assumed as in Langevin dynamics of the coarse-grained model described above, but the dihedrals were the only degrees of freedom in the RISM. Each dihedral was assumed to undergo jumps between three equivalent states 1, 2, 3, with the same value of the jumping rate coefficients, *k*_12_ = *k*_21_ = *k*_23_ = *k*_32_ = *k*_13_ = *k*_31_ = *k*. The stochastic time evolution of each dihedral was computed using the standard kinetic Monte Carlo scheme. Further details are given in the SI.

## Results

### The transition from the barrier friction limit to the Rouse/Zimm regime

In Kuhn’s original argument, the effect of barrier friction on the global relaxation dynamics will become increasingly small as the chain becomes longer, because the barrier friction decreases with the increasing chain length (see Introduction) while the hydrodynamic friction increases. As a result, the global reconfiguration time *τ*_*r*_ approaches the limit where it obeys the Rouse or Zimm model and, accordingly, grows as a power law with the chain length *N* (cf. equation (1)), while the internal friction characteristic time *τ*_*i*_ remains independent of *N*. The transition between these limits can also be observed by studying the solvent viscosity dependence of *τ*_*r*_; however simulations of proteins^31^ indicate that the dihedral relaxation times show a weak, but non-negligible solvent viscosity dependence thus complicating deconvolution of the two effects. In a computational (as opposed to real) experiment, there is an alternative (and often easier) approach: keep the chain length fixed but vary the height of the hindered rotation barrier to control the dihedral rotation time. This latter approach was used in ref. 30 to prove that reduced dihedral angle barriers lead to enhanced conformational mobility of an unfolded protein under poor solvent conditions.

To systematically study this transition in the absence of complications resulting from sequence effects and to avoid the high computational cost of simulating long polypeptide chains at high solvent viscosities, we first resorted to coarse-grained simulations. Specifically, we used Langevin dynamics simulations of a C_α_-only coarse-grained model of a random-coil homopeptide, as described earlier^26, 50–53^. Explicit treatment of water molecules is, however, required in order to reproduce the correct viscosity dependence of the dihedral dynamics^31^; thus solvent viscosity effects cannot be adequately captured in this treatment. Nevertheless, the relationship between the overall reconfiguration time and *τ*_*dih*_ is easily investigated in this approach by varying chain length and the magnitude of the dihedral barrier.

**Figure 1:**
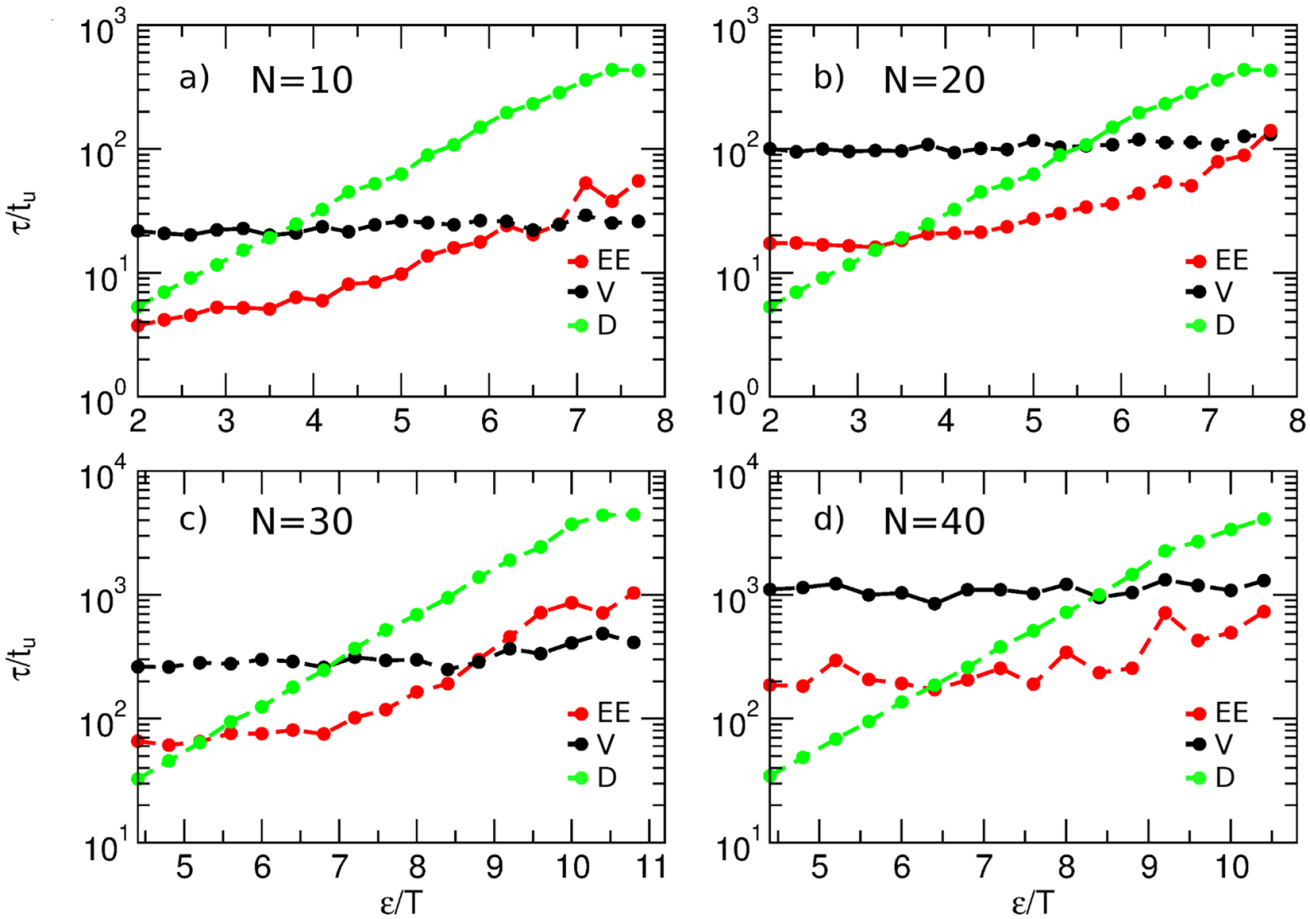
The end-to-end distance (EE), end-to-end vector (V), and dihedral angle relaxation times (D) as a function of the dihedral barrier in coarse-grained peptides of varied length *N*.

The dihedral energy landscape of our model peptide is controlled by a force-field term *V*_*dih*_ = *ε*(1 − cos3*φ*)/2, which has a 3-fold symmetry, and whose minima are separated by barriers equal to *ε*. For chains with *N* = 10, 20, 33, and 66 monomers, we computed the relaxation times of the end-to-end vector **R**, end-to-end distance *R* = |**R**|, and each dihedral angle as a function of *ε* (Fig. 1). The relaxation time of each of these quantities was defined as

$$\tau ={\int }_{0}^{\infty }C(t)dt / C(0),$$

where *C*(*t*) is the autocorrelation function given by

$$C(t)=\langle{\rm{\Delta }}X(0){\rm{\Delta}}X(t)\rangle,{\rm{\Delta}}X=X-\langle X \rangle$$

for *X* = **R** or *R* and

$$C(t)=\langle cos[\phi (t)-\phi (0)]\rangle$$

for a dihedral *φ*. Note that, unless *C*(*t*) is an exponentially decaying function, there is no unique definition of the associated characteristic time *τ* - the above heuristic definition is, however, commonly used. We further note that the dihedral relaxation time depends on the location of the dihedral within the chain, with the dihedrals belonging to the chain extremities moving faster than those in the middle (see SI, Fig. S2); however, the difference was always less than a factor of two. Figure 1 reports the average dihedral relaxation times, with the dihedrals belonging to the chain extremities (2 outer dihedrals at each chain end) excluded from the average.

Consistent with the picture of activated barrier crossing, the dihedral relaxation time *τ*_*dih*_ increases exponentially with *ε* (Fig. 1, green); it is further found to be independent of chain length *N*. The end-to-end distance relaxation time *τ*_*EE*_ (Fig. 1, red) is greater than *τ*_*dih*_ (except for the shortest peptide considered) and nearly independent of the dihedral barrier at low values of *ε*. In contrast, at high values of *ε*, *τ*_*EE*_ becomes shorter than *τ*_*dih*_; moreover, we observe direct proportionality between the two quantities, *τ*_*EE*_ ∝ *τ*_*dih*_ (i.e., the green and the red lines become parallel in the logarithmic plot of Fig. 1). This indicates that the barrier friction limit is attained, where the chain remains essentially frozen until a dihedral changes, and where, as a result, the timescale *τ*_*dih*_ controls the global relaxation dynamics as measured by *τ*_*EE*_.

Conversely, the low *ε* limit is associated with the Rouse model behavior (note that, since our Langevin dynamics simulations did not include hydrodynamic interactions, we are comparing the results with the Rouse rather than the Zimm model). In support of this conclusion, the end-to-end vector relaxation time *τ*_*V*_ measured in this limit (Fig. 1, black) is close to the slowest relaxation time estimated using the Rouse model (given by equation (1), where the translational diffusion coefficient is given by *D*_*tr*_ = *k*_*B*_ *T*/(*Nξ*_0_) and *ξ*_0_ is the monomer friction coefficient), as expected to be the case for the Rouse model^25, 42^ – see SI, Table S1.

Moreover, at low values of *ε*, the vector relaxation time *τ*_*V*_ is longer than *τ*_*EE*_ by a factor of ~3 (see SI, Table S1), a result consistent with previous theoretical predictions for the Rouse chain^17^. However, as the dihedral barrier increases, *τ*_*V*_ shows only a weak increase and eventually becomes shorter than *τ*_*EE*_. This result is easy to understand: even if all the dihedral angles are frozen, the chain can still rotate, with its rotational relaxation controlled by the solvent friction (while the end-to-end distance does not significantly fluctuate). We note that the Rouse model with internal friction fails to properly account for the rotational dynamics of the chain and predicts the end-to-end vector relaxation to be determined by internal friction in this limit^42^.

**Figure 2:**
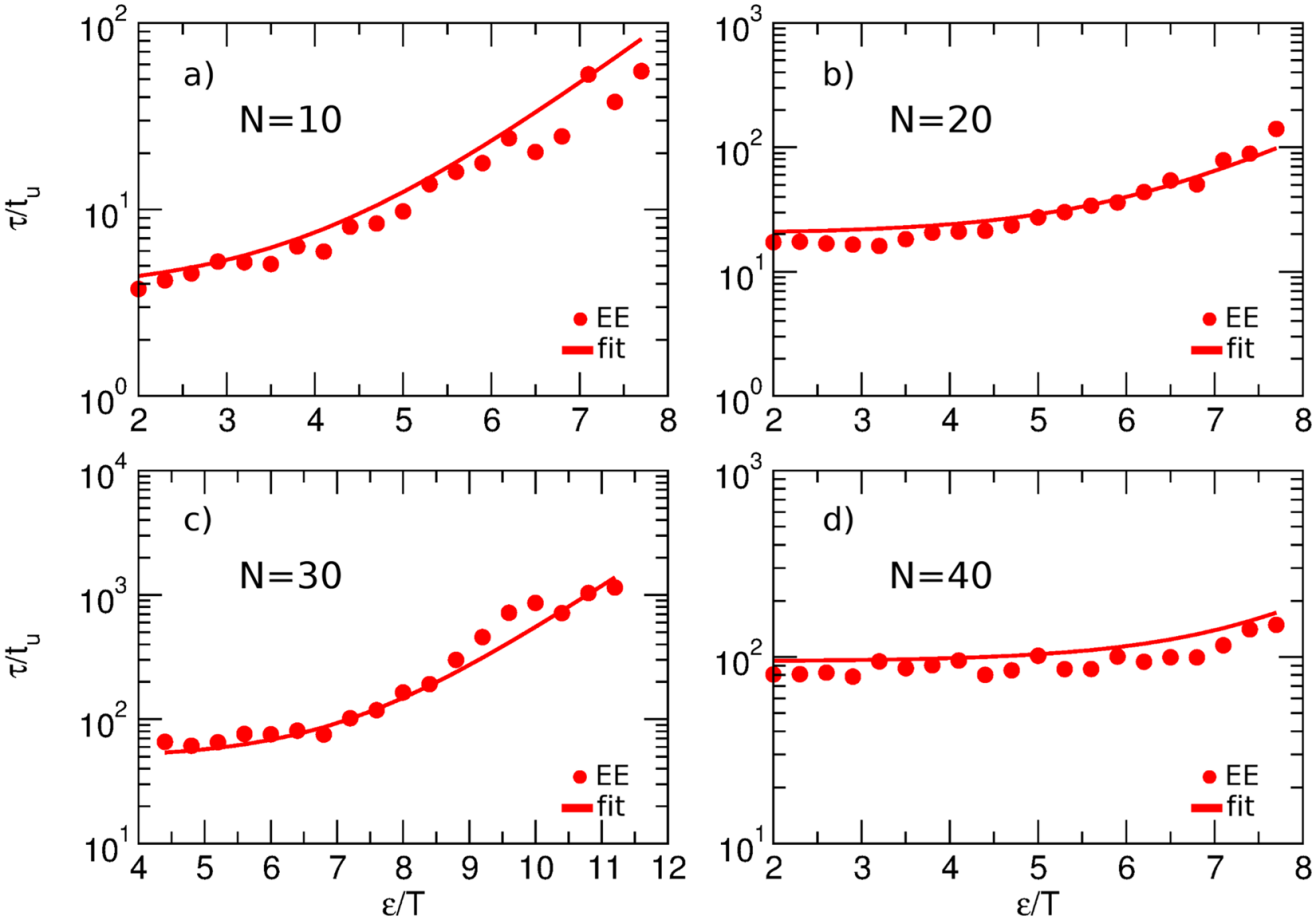
End-to-end distance relaxation time (EE), as a function of the dihedral barrier, for chains of different length. Lines represent the global fit of the data by equation (2), with *a* = 0.26 and *b* = 0.15.

Since most experimental measurements of internal chain dynamics probe absolute distances between different chain segments and are insensitive to orientational dynamics, we now focus on the end-to-end distance relaxation time *τ*_*EE*_. We have discussed above that this time is proportional to the Rouse time *τ*_*R*_ (defined here as the longest relaxation time of a Rouse chain, equation (1)) at low values of the barrier to hindered rotations or for long chains; it is proportional to the dihedral relaxation time *τ*_*dih*_ in the opposite limit. Hence, it is natural to try the simple linear combination of the form

2 $${\tau }_{EE}=a{\tau }_{R}+b{\tau }_{dih},$$

to interpolate between the two limits. Indeed, equation (2) is found to globally fit the data well for all chain lengths and dihedral barriers, as shown in Fig. 2. Here the optimal values of the parameters, *a* = 0.26 and *b* = 0.15, describe the data for all peptide lengths and all dihedral barriers. Note that the value of *a* is comparable to the value expected using analytic estimates for a Rouse chain. Indeed, an analytic approximation due to Portman^17^ predicts that, in the long-time limit, the end-to-end distance autocorrelation function for a Gaussian chain reaches its asymptotic limit at twice the rate at which the end-to-end vector autocorrelation function decays, implying *τ*_*EE*_ ≈ 0.5 *τ*_*V*_. Combined with the relationship^42^ *τ*_*V*_ ≈ 0.8 *τ*_*R*_, this yields *τ*_*EE*_ ≈ 0.4 *τ*_*R*_. Given nonexponentiality of the correlation functions, however, the precise definition of the relaxation time affects the expected value of the proportionality coefficient between the two timescales.

### Implications for experimental studies of internal friction

While here we have been able to observe the transition from the Rouse regime to a barrier-controlled regime by varying chain length and the magnitude of the dihedral barrier, those are not common experimental variables. Rather, two most common ways to observe deviations from solvent-dominated dynamics are to study how the protein reconfiguration time depends on the solvent viscosity *η* ^27^ and how the reconfiguration time of a shorter segment of the entire protein depends on its length^26^.

Empirically, experimental viscosity dependence of *τ*_*EE*_(*η*) is often close to linear^27^, and so the zero viscosity intercept of this dependence provides an operational definition of internal friction. Moreover, this linear dependence suggests additivity of the contributions from internal friction (equal to the zero-viscosity intercept) and the solvent controlled friction (which is directly proportional to viscosity). This is consistent with the additivity of the barrier friction and the Rouse-Zimm-type friction effects predicted by Equation (2). It is, however, not possible to directly compare the experiments with coarse simulations for two reasons. First, since Langevin dynamics simulations in the overdamped regime were used to obtain Eq. 2, both *τ*_*R*_ and *τ*_*dih*_ are proportional to the solvent friction within the coarse model employed here. Second, if the dihedral relaxation time *τ*_*dih*_ is viscosity dependent, the zero viscosity intercept of *τ*_*EE*_(*η*) predicted by Eq. 2 cannot be simply equated with the term *bτ*_*dih*_.

Indirect comparison between predictions of Eq. 2 and experimental studies is still possible if we consider additional information coming from atomistic studies. Specifically, atomistic simulations from the Best group^31^ and our own work^30^ show only a weak solvent viscosity dependence of *τ*_*dih*_. Assuming the validity of equation (2) as applied to real proteins, then, the viscosity dependence of the observed reconfiguration time should mostly result from its first term. In contrast, the internal friction time *τ*_*i*_, identified with the second term of equation (2), should not show significant viscosity dependence, which is consistent with experimental observations^27^.

Identification of the dihedral relaxation time with the internal friction time *τ*_*i*_ is further supported by a more detailed analysis of intra-chain dynamics. For example, while at low values of the dihedral barrier the reconfiguration time between the mid-segment of the chain and its end is shorter than that between the chain ends, in accord with the predictions of the Rouse and Zimm models^26^, the two times converge as the dihedral barrier increases (See SI, Fig. S3). This observation agrees with the prediction of RIF that, at high internal friction, relaxation modes of different wavelengths have approximately the same relaxation time *τ*_*i*_^27^. Likewise, it agrees with the argument (see the Introduction) that a chain whose dynamics is dominated by barrier friction exhibits a single, segment-length-independent timescale.

### Concerted or uncorrelated dihedral rotations?

Having described the transition between the solvent- and barrier-friction dominated regimes, we now focus on the barrier friction limit and try to further elucidate the microscopic origins of equation (2). In particular, we would like to know whether the rotations of different dihedrals tend to occur independently of one another or are correlated, and whether such correlations (or their lack) can be deduced from the observed end-to-end dynamics. Independent dihedral rotations (particularly those occurring near the middle of the polypeptide chain) would involve large swinging motion of two parts of the chain, thereby entailing both steric clashes and high solvent friction. But while this argument is often used to justify correlated dihedral hops that result in more localized, crankshaft-type motions of the polymer, such concerted changes of the dihedrals must require higher activation barriers^44^ – the outcome of this tradeoff between lower friction but higher activation barrier is unclear in advance and may depend on chain length and the magnitude of the dihedral barrier.

To understand the connection between local dihedral changes and the global dynamics, it is helpful to first consider the one-dimensional toy model introduced by Hall and Helfand^54^. In this model, the polymer is a one-dimensional chain of *N* bonds, with each bond having a length jumping between two possible values, *l*_−_ and *l*_+_. These jumps mimic the dihedral rotations in 1D; the result of each jump is a change of the total end-to-end distance by ±Δ*l* ≡ ± |*l*_+_ − *l*_−_|. Let us further assume that the jumps of each bond can be described by a first-order kinetic scheme, ${l}_{-}k\mathop{\rightleftarrows }\limits_{k}{l}_{+}$. The total chain length undergoes a one-dimensional random walk with a step Δ*l* and with an average frequency *v* = *kN*, since *N* bonds are each jumping independently. At short enough times, the mean square displacement of such a random walker is given by $\langle{\rm{\Delta }}{R}^{2}(t)\rangle={\rm{\Delta }}{l}^{2}\nu t={\rm{\Delta }}{l}^{2}kNt$. Equating this with 2*Dt* defines an effective end-to-end diffusion coefficient *D* = *N*Δ*l* ^2^ *k*/2 = (1/4)*N*Δ*l* ^2^/*τ*_*bond*_, where *τ*_*bond *_= (2*k*)^−1^ is the relaxation time of a single bond. Importantly, this diffusion coefficient is proportional to chain length *N*.

After a sufficiently long time, the chain length will adopt a Gaussian distribution with a mean *N*(*l*_+_ + *l*_−_)/2 and a variance *ρ* ^2^ = *N*Δ*l* ^2^/4. The relaxation time of the end-to-end distance can then be estimated as a time it takes the random walk to travel the distance *ρ*:

3 $${\tau }_{r}=\frac{{\rho }^{2}}{2D}={\tau }_{bond} / 2$$

This result is a one-dimensional analog of the barrier friction limit, with the bond relaxation time being analogous to *τ*_*dih*_. Since this argument can be applied not only to the entire chain but also to any of its segments, this simple calculation provides yet another explanation of why single, segment independent relaxation timescale emerges from microscopic conformational dynamics in this limit.

Extension of these arguments to 3D is somewhat tricky. Unlike the 1D case, where the step size Δ*l* along the end-to-end distance direction is fixed, the change of this distance as a result of a change in a particular dihedral depends both on the dihedral and on the current chain configuration. To make progress, let us assume that one can characterize the random walk along *R* by an average value of the step size Δ*l* instead. The frequency of individual dihedral hopping *v* is equal to the inverse of the mean dwell time in one of the dihedral states. If we represent the kinetics of an individual dihedral by a 3-state system, with a rate of jumping between adjacent states equal to *k*, then this mean dwell time is (2*k*)^−1^ and so *v* = 2*k* (the factor of two comes from the fact that there are two possible states that a dihedral can jump to). The effective diffusion coefficient is then given by *D* = *N*Δ*l* ^2^ × (2*k*/2). The dihedral relaxation time *τ*_*dih*_ for our 3-state model can be estimated as the inverse of the lowest eigenvalue of the corresponding 3 × 3 rate matrix and is equal to *τ*_*dih*_ = (3*k*)^−1^. This gives *D* ≃ (1/3)*N*Δ*l* ^2^/*τ*_*dih*_. The relaxation time of the entire chain can now be estimated as the time to diffuse the distance comparable to the root-mean-square end-to-end distance, equal to $\rho \sim {(L{l}_{k})}^{1 / 2}={n}_{k}^{1 / 2}{l}_{k}$, where *L* is the polypeptide contour length, *l*_*k*_ is the length of its Kuhn segment, and *n*_*k*_ = *L*/*l*_*k*_ is the number of the Kuhn segments in the chain. This gives

4 $${\tau }_{r}\simeq \frac{{\rho }^{2}}{2D}=\frac{3{l}_{k}\sigma }{2{\rm{\Delta }}{l}^{2}}{\tau }_{dih},$$

where *σ* is the peptide bond length. There are two possibilities now. In the case of crankshaft-type moves where a pair of dihedrals change simultaneously such that only a local chain segment is affected while there is no global rearrangement of the entire chain, Δ*l* is a geometry dependent number that would be typically much smaller than *ρ* and comparable to the Kuhn length. For example, assuming Δ*l* = *l*_*k*_ we find *τ*_*r*_ = (3/2)*τ*_*dih*_(*σ*/*l*_*k*_). The ratio *l*_*k*_/*σ* is the number of peptide bonds within one Kuhn segment, whose value is ~3 for the peptides studied (see the SI, Table S1). Thus the fact that *τ*_*r*_ is shorter than *τ*_*dih*_ and so the factor *b* in equation (2) is less than one is naturally explained within this picture.

The second possibility arises where all dihedrals change independently. When a single dihedral changes its value (and assuming no other relaxation mechanisms present), the ensuing large-scale pivoting motion results in a distance change Δ*l* that is comparable to the dimensions of the chain itself. In this case, of course, the motion of end-to-end distance cannot be viewed as diffusion. A better model would be one where each dihedral rotation causes the chain to completely lose the memory of its end-to-end distance (over the time of a pivoting motion). The effective reconfiguration time, therefore, is comparable with the inverse frequency of dihedral transitions, which is equal to

5 $${\tau }_{r}={(2kN)}^{-1}=3{\tau }_{dih} / (2N)$$

The heuristic prediction that the barrier friction time is inversely proportional to the chain length in this case is verified explicitly in the SI (see Fig. S6) using a rotational isomeric state model of a polypeptide with independently changing dihedrals.

Since no such chain length dependence is observed in our simulations, we conclude that concerted dihedral motions dominate the barrier friction time, in accord with ref. 30. Similar analysis based on atomistic simulations leads to the same conclusion - see the following discussion.

We have also observed temporal correlations between sequence distant dihedrals directly in the following way. Consider a pair of dihedrals labeled, say, *i* and *j*. If the flipping of each dihedral is an independent Poisson process with a characteristic time ${\tau }_{dih}^{(i,j)}$, then the distribution of the time lag *t* between the flip of *i* and subsequent flip of *j* is exponential^55^, ${p}_{ij}(t)=[{({\tau }_{dih}^{(i)})}^{-1}+{({\tau }_{dih}^{(j)})}^{-1}]{e}^{-t[{({\tau }_{dih}^{(i)})}^{-1}+{({\tau }_{dih}^{(j)})}^{-1}]}$. In contrast, correlation between the two events will lead to deviations from this exponential distribution. Indeed, while the dynamics of each individual dihedral is well described by a Poisson process, the lag time distributions for the pairs of dihedrals that are close to one another in sequence deviates from exponential, showing positive correlation between their jump times (see the SI, Fig. S5). The correlation disappears at large sequence separation. This behavior is similar to the earlier findings of a finite correlation length in atomistic simulations of the cold-shock protein^30^.

### Dihedral dynamics vs. global dynamics in the cold shock protein and its short fragments

Having developed insights about the connection between global peptide dynamics and microscopic timescales associated with dihedral rearrangements, we next examine whether any of these carry over to atomistic models of proteins and polypeptides. To this end, we have used the already published data on the dynamics of the cold shock protein (CSP), simulated, at a fully atomistic level in explicit solvent, using two different force fields. One simulation^30^ uses a conventional force field, as described in the Methods section. This simulation will be referred to as CSP1. Recently, Piana *et al*.^56^ proposed to modify water dispersion interactions in order to achieve better agreement with experimental estimates of protein dimensions– we use one of their trajectories from ref. 56 (specifically, the one displaying the best agreement with the experimental single-molecule FRET data^27^) – we will refer to this as CSP2. In addition, we have performed explicit-solvent atomistic simulations of 8 short polypeptides, each 11 residues long. The first 6 were fragments of the cold-shock protein previously studied experimentally^27^ and via molecular dynamics simulations^30^. Our rationale for choosing these fragments of a well studied protein is to examine how changing the length of a polypeptide (from *N* = 66, which is the full length of CSP, to *N* = 11) affects the dynamics. In particular, the arguments presented in the Introduction and leading to the Kuhn barrier friction picture as well as to the ZIF/RIF models predict that the internal friction timescale *τ*_*i*_ would remain unchanged as a result of this length change; these predictions are, however, based on the idealized picture of highly localized conformational changes within homopolymer chains and are not rigorously justified. At the opposite extreme, a model where all the dihedrals change independently and where, consequently, a single dihedral jump may lead to a global rearrangement of the entire chain predicts that *τ*_*i*_ would become longer for such shorter peptides (see equation (5)), provided that the dihedral relaxation times stay the same. Of course, polypeptides of only a few Kuhn segments in length may not be adequately described by simple polymer models. Moreover, sequence-dependent effects should be significant if not dominant for such short peptides. In order to assess the role of such effects, we also performed all-atom simulations of an 11-residue peptide with the Gly-Ser repeat, a system that is often deemed to be a model random coil polypeptide. Finally, to study how the height of the dihedral barrier affects the dynamics, we studied the same Gly-Ser repeat sequence with its dihedral barrier reduced by a factor of 2. The simulation results are summarized in Table 1.

**Table 1:** Summary of the relaxation times measured for 8 short peptides and the cold shock protein (CSP) simulated using two different force fields.

Peptide	End-to-End Distance Relaxation Time (ns)	End-to-End Vector Relaxation Time (ns)	Average Dihedral Angle Relaxation Time (ns)	*D*_*tr*_ (cm^2^/s) (From MSD)
1–11	44.8	1.78	22.3	2.32e-6
12–22	52.1	1.57	20.6	2.62e-6
23–33	29.0	1.98	17.8	2.46e-6
34–44	10.3	2.12	21.2	2.23e-6
45–55	24.0	2.11	17.0	2.38e-6
56–66	76.0	1.66	44.9	2.71e-6
Gly-Ser	11.6	0.77	13.7	3.72e-6
Modified Gly-Ser	2.91	0.76	3.60	3.78e-6
CSP1^30^	38.2		29.1	
CSP2^56^	127		281	

*Each of the polypeptides showed a spectrum of dihedral relaxation times*, with dihedral autocorrelation functions decaying on timescales from ~0.1 ns to hundreds or even thousands of nanoseconds (See the SI, Fig. S41). Some of the dihedrals within the CSP fragments either stayed unchanged during the course of a 2-microsecond-long simulation or exhibited very few rotations, preventing us from getting a converged autocorrelation function and estimating the resultant relaxation time. As a result, estimation of individual dihedral relaxation times for all the angles is not possible even with a trajectory as long as 60 microseconds, let alone the 2-microsecond-long simulations of the shorter peptides. To improve the statistics in estimating autocorrelation functions of the dihedral angles, we have computed the mean autocorrelation function by averaging it over all dihedrals, as in ref. 30. From this function, a single, average dihedral relaxation time was estimated. These results are reported in Table 1.

#### The average dihedral relaxation times for CSP fragments were typically shorter (but of the same order of magnitude) than the full CSP1 simulated with the same force field

The longer timescales of rotational dynamics in the longer peptide presumably originate from stronger steric interactions^30^. The mean dihedral relaxation time for the modified Gly-Ser repeat with a lower dihedral barrier was a factor of ~4 faster, supporting the view that activated crossing of the dihedral barrier controls the timescale of dihedral dynamics. Somewhat unexpectedly, the dihedral relaxation time for CSP2 was found to be nearly an order of magnitude longer than for CSP1, despite the less compact CSP2 conformational ensemble^56^. This difference is presumably due to the difference in the force fields used in the two simulations.

#### The simulated peptides are in the barrier friction regime

The end-to-end vector relaxation times *τ*_*V*_ for all of the short peptides are much (3–10 times) shorter than their end-to-end distance relaxation times *τ*_*EE*_. At the same time, all values of *τ*_*V*_ are further within a factor of 3 from the time *τ*_*RZ*_ estimated from equation (1), where the translational diffusion coefficients *D*_*tr*_ for each peptide were inferred directly from the simulations (Table 1). Recall from the above discussion that we expect *τ*_*V*_ to be a factor of ~3 *longer* than *τ*_*EE*_ in the limit where Rouse/Zimm-type friction dominates. These observations show that all of the peptides are far away from this limit and that internal dynamics of dihedrals controls the relaxation of their end-to-end distance.

#### The end-to-end distance relaxation is comparable to (and correlated with) the dihedral relaxation time

**Figure 3:**
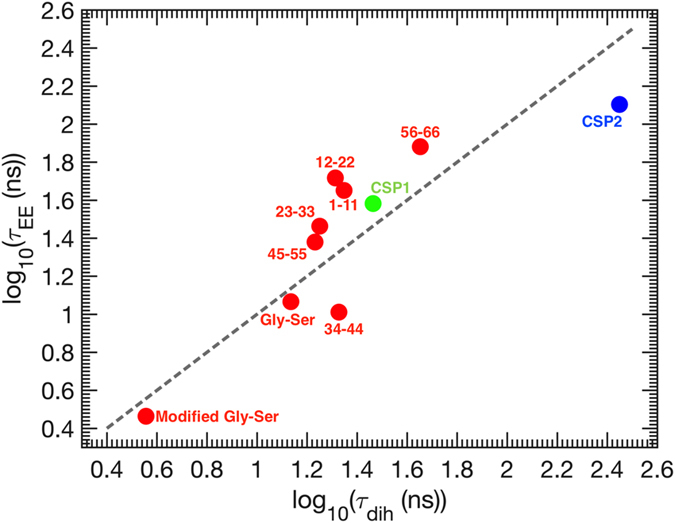
Dihedral relaxation time vs. end-to-end distance relaxation time.

Furthermore, both *τ*_*dih*_ and *τ*_*EE*_ are proportionally shorter for the CSP fragments as compared with CSP1 (Fig. 3). Let us recall that the Kuhn barrier friction model and the related RIF/ZIF models (in the limit of high internal friction) predict independence of the global relaxation timescale on chain length; this prediction, however, is contingent on the independence of the dihedral barrier crossing time on chain length, which is not the case here. Since both *τ*_*dih*_ and *τ*_*EE*_ increase when chain length increases from *N* = 11 to 66, this, in fact, is consistent with Kuhn’s barrier friction picture. Moreover, this behavior implicates concerted dihedral motions as controlling the end-to-end distance dynamics, since *τ*_*EE*_ is expected to become shorter with increasing *N* in the case where dihedrals change independently (cf. equation (5)).

Consistent with our coarse-grained simulations, which predict proportionality between *τ*_*dih*_ and *τ*_*EE*_ in the barrier friction limit (equation (2)), peptides simulated atomistically also show direct proportionality between these two timescales (Fig. 3). The proportionality factor *b* is, however, different, being close to 1 (Fig. 3), as opposed to *b* = 0.15 found in coarse-grained simulations. This difference between atomistic and coarse-grained models may be purely geometrical in nature: recall that the coarse model employed in our work (see the Methods section) consists of alpha-carbons only and thus has only one dihedral angle per amino acid residue. In contrast, microscopic description of a real polypeptide involves two dihedrals (*φ* and *ψ*) per residue. Notwithstanding this difference, the proportionality factor between *τ*_*dih*_ and *τ*_*EE*_ is found to be independent of the length of the chain in both cases. This key observation supports the Kuhn-type barrier friction view, with a single, chain length independent reconfiguration timescale (see Introduction).

#### End-to-end dynamics of polypeptides is subdiffusive, reflecting memory effects and deviations from RIF/ZIF predictions

A common description adopted by most experimental studies that probe relative motion of polypeptide chain segments is that of one-dimensional diffusion in an effective potential, which is determined by the entropic elasticity of the chain^8, 57–61^. Although it is known that the monomer motion of a polymer chain is not simple diffusion^15, 25, 62^, an effective diffusion coefficient that depends on the time and/or length scale of the process of interest still can sometimes be introduced to describe this process^15, 58, 62–64^. The monomer motion of a Rouse chain, in particular, is subdiffusive at intermediate timescales that are longer than the monomer relaxation timescale but shorter than the Rouse time, with the mean square monomer displacement scaling as $\langle{\rm{\Delta }}{R}^{2}(t)\rangle\propto {t}^{0.5}$. In the high internal friction limit, in contrast, the Rouse model with internal friction predicts simple diffusive dynamics^42^.

The possibility of dihedral rotations associated with large-amplitude monomer displacements introduces a different kind of non-diffusive dynamics. Consider, for example, the (already discussed) model where the end-to-end distance *R* loses the memory of its previous value every time a dihedral rotation occurs. The statistics of dihedral jumps is Poisson, with the average number of jumps per unit time equal to *ν*. Given no memory of the previous configuration after a jump, the new value of *R* is simply a random number drawn from the equilibrium probability distribution *p*(*R*). Let *R*_1_ be the end-to-end distance at time *t* = 0 and *R*_2_ at time *t*. The probability that *R*_2_ = *R*_1_ is equal to the probability that a jump did not happen during the time interval *t*, which is *e* ^−*vt*^ for a Poisson process. The probability that *R*_2_ is different from *R*_1_ is thus (1 − *e* ^−*vt*^). Averaging over these two possibilities (the jump did not or did happen) and taking advantage of the statistical independence of *R*_1_ and *R*_2_ in the case where the jump did happen, one obtains

$$\langle{\rm{\Delta }}{R}^{2}(t)\rangle={e}^{-\nu t}\langle{({R}_{1}-{R}_{1})}^{2}\rangle+(1-{e}^{-\nu t})\langle{({R}_{2}-{R}_{1})}^{2}\rangle=(1-{e}^{-\nu t})[\langle{R}^{2}\rangle-{\langle R\rangle}^{2}]\propto t$$

at short times. Therefore, the dynamics of *R* appears to be simple diffusion. Examination of higher-order moments, however, reveals that it is not! Indeed, the value of any moment $\langle{\rm{\Delta }}{R}^{2n}(t)\rangle$ is proportional to the probability (1 − *e* ^−*vt*^) of at least one dihedral transition during the time *t* and, therefore, exhibits exactly the same time dependence. In contrast, for the simple diffusion we have $\langle{\rm{\Delta }}{R}^{2n}(t)\rangle=(2n-1)!!{\langle{\rm{\Delta }}{R}^{2}(t)\rangle}^{n}\propto {t}^{n}$, so that the ratio $\langle{\rm{\Delta }}{R}^{2n}(t)\rangle / {\langle{\rm{\Delta }}{R}^{2}(t)\rangle}^{n}$ stays constant.

**Figure 4:**
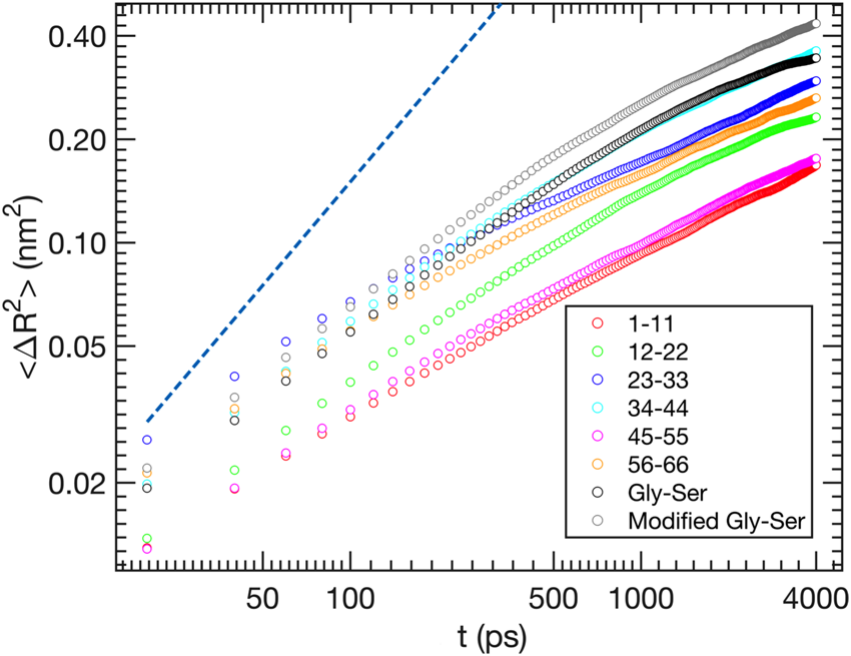
The end-to-end dynamics of all of the peptides studied here is subdiffusive, with mean square displacement $\langle\Delta {R}^{2}\rangle$ scaling as ${t}^{0.4-0.7}$ at short times *t*. For comparison, the dashed line shows the behavior expected for normal diffusion.

Examination of the end-to-end trajectories yields, at *t* → 0, $\langle{\rm{\Delta }}{R}^{2n}(t)\rangle={t}^{n\alpha }$ with α ≃ 0.4–0.7 for both the atomistic and coarse-grained simulations (Fig. 4; only atomistic data are shown). This, again, supports the picture where independent dihedral rotations leading to large changes in the end-to-end distance are improbable.

A more surprising finding is that the values of *α* are significantly less than the value (of 1) expected for simple diffusion both in the Rouse limit (where this is expected) and in the barrier friction limit. This indicates a subdiffusive, and, therefore, non-Markov process with prominent memory effects and contradicts the RIF or ZIF, which predict subdiffusion in the Rouse/Zimm limit but diffusive dynamics in the barrier friction limit^42^.

**Figure 5:**
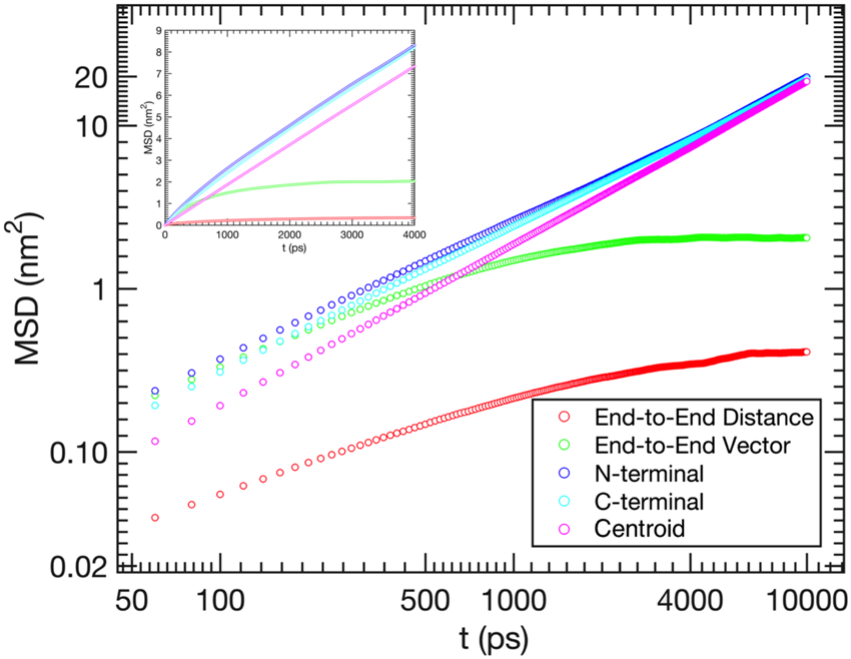
Mean square displacement of the end monomers of the Gly-Ser repeat construct grows nearly linearly at short times (*α* ≈ 0.9), approaching the strictly linear dependence and converging with the mean-square displacement of the peptide’s centroid (defined as the average position of the peptide’s alpha carbons) at long times. Despite this almost purely diffusive motion of the peptide’s ends, the relative distance *R* between them undergoes subdiffusive motion. The much lower values of $\langle\Delta {R}^{2}\rangle$, as compared to the monomer mean square displacements, indicates that, at short times, the latter are dominated by rotations of the polymer chain.

To gain further insight into peptide dynamics in the barrier friction regime, we computed the mean square displacements of the end monomers of the Gly-Ser construct as $\langle{\rm{\Delta }}{{\bf{R}}}_{i}^{2}(t)\rangle$, where **R**_*i*_(*t*) is now a vector describing the position of the first (*i* = 1) or last (*i* = 11) alpha-carbon of the peptide (Fig. 5). At short time *t* it is found to grow slightly slower than linearly (*α* ≈ 0.9), while approaching a strictly diffusive behavior and converging with the linear dependence for the mean square displacement of the peptide’s centroid. In other words, the movement of the end monomers is nearly diffusive even at short times, in contrast to the predictions of the Rouse or Zimm models but consistent with those of RIF and ZIF in the high internal friction limit.

Since the relative distance measured between two statistically uncorrelated diffusing particles also undergoes simple diffusion, the much stronger deviation from simple diffusion observed for the end-to-end distance indicates that the displacements of the peptide ends are strongly correlated at short times. The source of such correlations is easily understood (albeit not explaining the subdiffusive behavior *per se*): when the time *t* is much shorter than the dihedral relaxation times, the displacements of the peptide atoms are mostly due to its overall rotation. Indeed, at short times, the mean square change in the end-to-end distance, $\langle{\rm{\Delta }}{R}^{2}\rangle$, is much smaller than the corresponding changes in the positions of its end monomers. Moreover, the mean square change in the end-to-end *vector*, $\langle{\rm{\Delta }}{{\bf{R}}}^{2}\rangle$, is much greater than $\langle{\rm{\Delta }}{R}^{2}\rangle$, again indicating that, at short times, the end-to-end vector mostly rotates without changing its magnitude. It is important to note that RIF and ZIF do not describe this rotational dynamics correctly^42^ in the internal friction dominated limit: they make the unphysical prediction that the rotational relaxation time is independent of solvent hydrodynamics and equal to *τ*_*i*_.

## Discussion

The subject of internal friction or viscosity has been extensively debated and never satisfactorily settled in the polymer physics of 1970–90’s^24, 35–37, 40, 41^, even for relatively simple polymeric systems such as hydrocarbons. Different physical mechanisms were invoked to explain internal friction effects and a number of distinct polymer models were proposed. In light of these complications one may wonder if a simple polymer-theoretical description is at all possible, especially in the case of unfolded proteins, whose energetics, involving hydrogen bonding, hydrophobic interactions, and other mechanisms^65^ is more complicated. Our study, while revealing limitations of simple models, also points toward near universal features exhibited by protein dynamics. One surprising finding is the subdiffusive, rather than diffusive monomer motion. While such breakdown of the simple diffusion model has been predicted, e.g., on the basis of the Rouse model^15, 62, 64^, internal friction effects treated using the Rouse model with internal friction (RIF) were expected to restore diffusive dynamics^42^ in the high internal friction limit, a prediction that is at odds with the present study. A possible explanation of this discrepancy is the fact that RIF, being essentially a one-dimensional model, does not adequately describe bond rotation. Indeed, it predicts internal friction effects, when they are dominant in the global reconfiguration dynamics, to dominate the rotational dynamics of the chain as well – this prediction is clearly absurd as even when all the internal degrees of freedom of the chain are frozen (i.e., infinite internal friction) it can still undergo rotational relaxation entirely controlled by the solvent hydrodynamic friction. Since the end-to-end distance of the chain changes in response to three-dimensional motion of the internal chain segments, it is conceivable that RIF would be inaccurate in describing the dynamics of the chain’s end-to-end distance.

At the same time, RIF (and the related ZIF) captures other aspects of protein dynamics. For example, consistent with these models, high internal friction leads to near-degeneracy of the relaxation timescales, where internal chain segments reconfigure on the same time scale as the entire chain^27, 30^ (see Fig. S3).

Moreover, in the case of protein dynamics within a coarse-grained model we see that the global reconfiguration obeys a simple RIF-like relationship, equation (2), provided that the internal friction time *τ*_*i*_ is identified, to within a chain-length-independent proportionality factor, with the dihedral relaxation time *τ*_*dih*_. Equation (2) provides a simple interpolation between the regime where the solvent friction dominates, achieved when the chain is sufficiently long, and the barrier friction regime, where the reconfiguration dynamics is controlled by dihedral rotations. Equation (2) is also consistent with atomistic simulations of peptides and proteins, although in the latter case we have only been able to explore the barrier friction limit.

Our study further supports the view that dihedral rotations in a polypeptide are concerted^30^. The most direct evidence of this comes from the correlation between the times at which the dihedrals that are close in sequence undergo rotations – see Fig. S5. Note that similar correlation was found in atomistic studies^30^. Further, indirect (but experimentally testable) evidence comes from the weak chain length dependence of the reconfiguration time (in the barrier friction limit), which contradicts chain-length dependent reconfiguration times predicted by a model where the dihedrals change independently. Finally, the subdiffusive character of the end-to-end distance dynamics observed in all of our simulations also contradicts the independent dihedral jump picture.

Our study shows that measurements of internal friction (such as the ones reported in ref. 27) provide information about the timescales of microscopic motion within the chain, specifically, its dihedral relaxation. But a key question remains to be answered: what properties of the polypeptide chain determine those timescales? Why do different proteins of comparable length and at the same conditions display different values of the internal friction time *τ*_*i*_ and, hence, different timescales of dihedral dynamics? Furthermore, what determines the length scale over which dihedral flips are correlated? A parallel can be made here with the problem of first-principles prediction of the dry friction coefficients, which is still an open issue. Experiments, atomistic simulations, and theoretical efforts will be required to further elucidate the microscopic basis of internal friction.

## Electronic supplementary material

SUPPLEMENTARY INFO
