# Supplementary material for: Theoretical and computational validation of the Kuhn barrier friction mechanism in unfolded proteins
Source: Sci Rep. 2017 Mar 21;7:269. doi: 10.1038/s41598-017-00287-5 (PMC5428071; doi:10.1038/s41598-017-00287-5)
Supplement: Supplementary file 1 — SUPPLEMENTARY INFO [file 41598_2017_287_MOESM1_ESM.pdf]

Supplementary Information

Theoretical and computational validation of the Kuhn barrier friction mechanism in unfolded proteins.

Stanislav M. Avdoshenko[1,4], Atanu Das[2], Rohit Satija[2], Garegin A. Papoian[3], and Dmitrii E. Makarov[1,2,*]

[1]Institute for Computational Engineering and Sciences, University of Texas at Austin, Austin, Texas 78712,

[2]Department of Chemistry, University of Texas at Austin, Austin, Texas 78712

[3]Department of Chemistry and Biochemistry, University of Maryland, College Park, MD 20742, USA,

[4]Current address: Leibniz Institute for Solid State and Materials Research (IFW Dresden), Helmholtzstraße 20, 01069, Dresden, Germany.

*Correspondence and requests for materials should be addressed to D.E.M.

(Email: makarov@cm.utexas.edu)

1. Simulations of the coarse grained model

Systems and parameterization. A coarse-grained C_a-only homopeptide model similar to those described earlier[1-5] represents each amino acid residue as a single bead and employs a 3-letter alphabet for the amino acid sequence, consisting of hydrophobic, neutral, and polar beads. To describe the unfolded polypeptide, sequences of various lengths (*N*=10, 20, 30, 40) consisting entirely of neutral beads were used. All calculations were done using an in-house Langevin code reported elsewhere[6,7].

The following parameters were used to specify the system's energetics and dynamics: The results are given in terms of dimensionless units, with the thermal energy *T* setting the energy units, the peptide bond length σ defining the units of length, and the parameter $t_u = \left(\sigma^2 / mT\right)^{1/2}$, *m* being an effective monomer mass, setting the units of time (t_u later in the SI text and figures). Chain connectivity was accounted for by a harmonic interaction potential acting between adjacent monomers, $V_{bond}(r) = k_{bond}(r-\sigma)^2/2$, with $k_{bond} = 100T/\sigma^2$, and a bending potential of the form $V_{bend}(\theta) = k_\theta(\theta-\theta_0)^2/2$ with $k_\theta = 20T/rad^2$ and θ_0 $=105^0$ enforced proper peptide bond geometry. Interactions between non-bonded monomers were described by a repulsive potential of the form $V_{nonb}(r) = 4T(\sigma/r)^{-12}$. The dependence of the energy on each of the dihedral angles φ was described by the potential $V_{dih}(\varphi) = \varepsilon(1-\cos 3\varphi)/2$, with the height of the dihedral barrier, ε, varied to study how the dihedral energy landscape affects the peptide dynamics. The dynamics of the chain was governed by the Langevin equation with a friction coefficient $\xi = 2\left(\frac{\sigma^2}{mT}\right)^{-1/2}$; the Langevin equation was integrated using the Verlet algorithm with a time step of $\Delta t = 0.02$.

Simulation Protocol. Starting with a linear initial structure of the chain, the system was equilibrated over 100000 simulation steps. Equilibration was followed by a production run including 55000000 steps. The peptide structure was saved every 1000 steps. This protocol was repeated for values of ε ranging from 2*T* to 8*T*, with an increment of $\Delta\varepsilon = 0.3T$. The probability distributions of the end-to-end distance *R* for each chain are shown in Figure S1, illustrating adequate sampling of the structural ensemble.

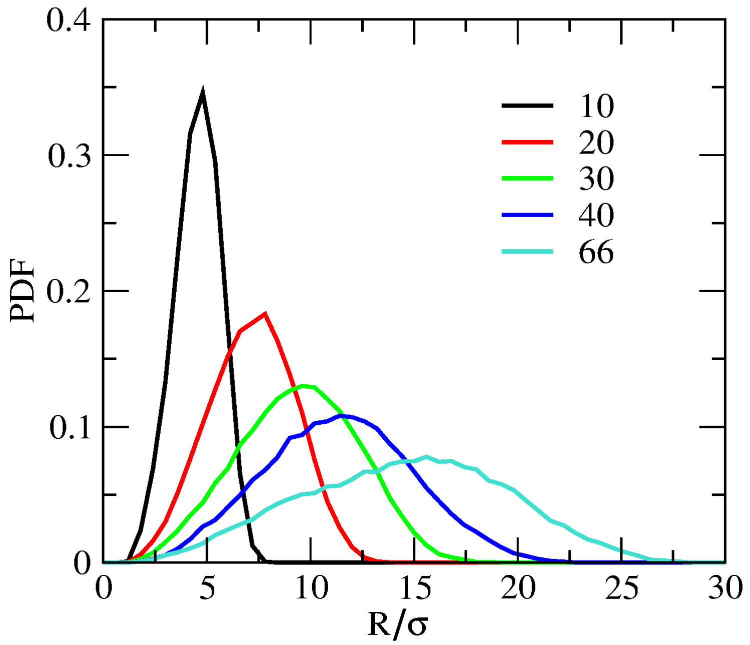

Fig. S1. Probability distributions of the end-to-end distance estimated from coarse-grained simulations for peptides of different length *N*.

Estimation of relaxation times. Characteristic relaxation times for the dihedral angles, end-to-end distance, and the end-to-end vector were estimated from the respective autocorrelation functions (ACF), as described in the main text. By fitting the decaying autocorrelation functions with an exponential function of the form $ACF(t) = a + ce^{-bt}$, the corresponding relaxation time was simply given by $\tau = 1/b$.

The dihedral relaxation times varied depending on the sequence location within the chain, with the dihedral belonging to chain extremities relaxing faster. This is illustrated in Fig. S2. In reporting the dihedral relaxation times averaged over the entre chain we thus removed such edge effects by excluding the contributions from the first and last 2 dihedrals in the chain.

In order to assess the spectrum of relaxation times within our coarse grained peptides, we compared the end-to-end distance relaxation times, t_{EE} , with the "end-to-middle" relaxation time t_{EM} estimated from the autocorrelation function of the distance between a chain end and the monomer located in its middle. In Rouse or Zimm theory (zero internal friction), t_{EM} is dominated by shorter-wavelength relaxation modes and, therefore, is shorter than t_{EE} [2]. In contrast, RIF and ZIF predict that all relaxation modes essentially become identical in the high friction limit, in which case t_{EE} should converge with t_{EM} [8]. Consistent with these predictions, t_{EM} is shorter than t_{EE} for low values of the dihedral barrier, but the two times become comparable as ε is increased (Fig. S3).

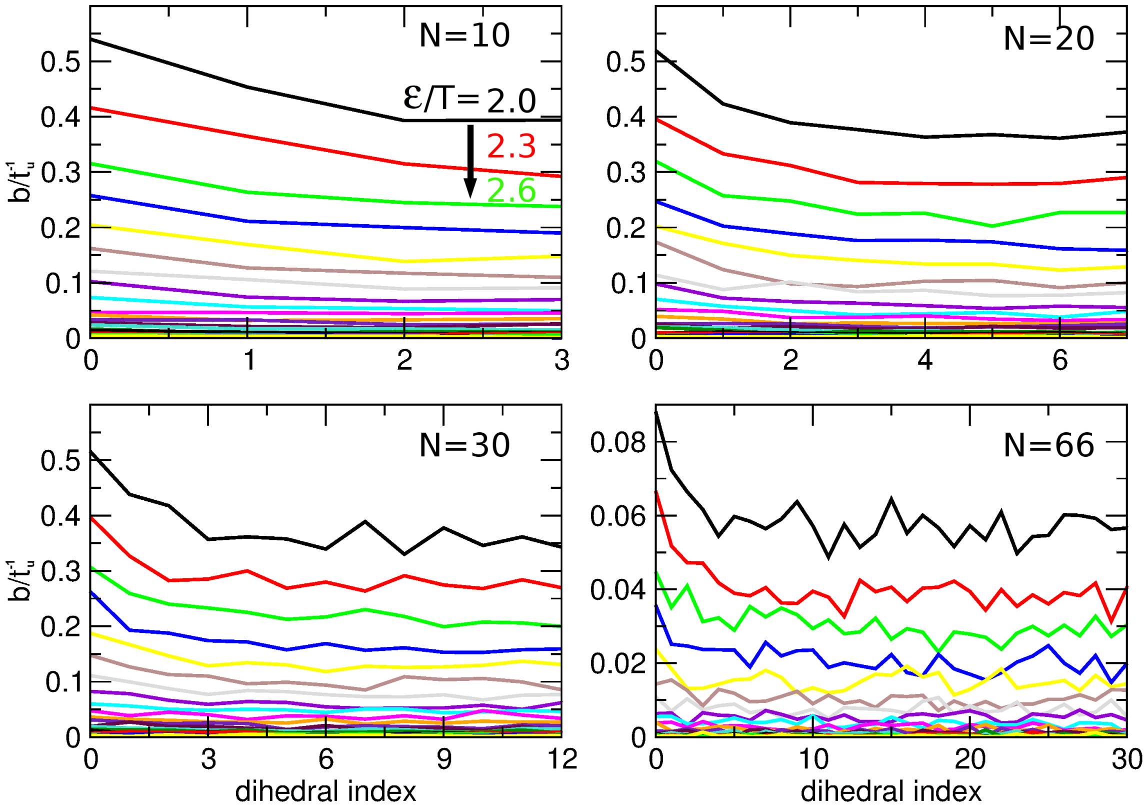

Fig. S2. Dihedral relaxation rate b (equal to $1/\tau_{dih}$) as a function of the sequence position for different values of the dimensionless rotational barrier, ε/T . Only one half of each chain is shown so that the highest value of the dihedral index corresponds to the middle of the peptide.

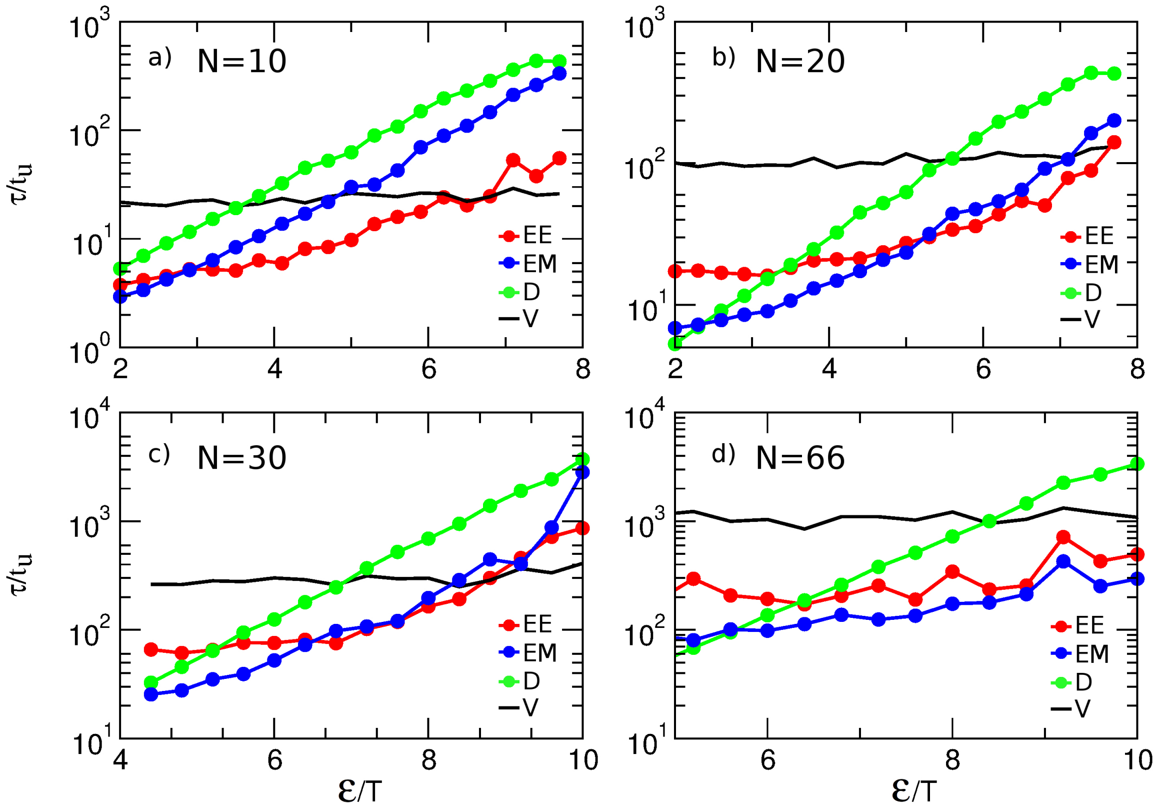

Fig. S3. Relaxation times of the end-to-end distance (EE), end-to-end vector (V), dihedral angle (D, averaged over dihedrals) and end-to-the-middle distance (EM) as a function of the dihedral barrier. Note that the end-to-end distance (EE) and the end-to-middle distance (EM) relaxation times converge as the dihedral barrier increases.

Validity of the Rouse picture for long chains and/or low dihedral barriers. As our coarse grained simulations did not include hydrodynamic interactions, we compare them with the Rouse model. In this model, the polypeptide is treated as a chain of n_k statistically independent Kuhn segments; the length of each is l_k. Since the original chain consists of N bonds of the length s, its total length is given by $L = N\sigma = n_k l_k$. Further assuming Gaussian statistics, we also have $\langle R^2 \rangle = n_k l_k^2$, allowing us to estimate the Kuhn length and the number of Kuhn segments as

$$l_K = \frac{\langle R^2 \rangle}{L} = \frac{\langle R^2 \rangle}{N\sigma}$$

$$n_k = \frac{L}{l_k} = \frac{L^2}{\langle R^2 \rangle}$$

Under the Rouse model assumption, the slowest relaxation time of the chain (i.e. the Rouse time) can be estimated as

$$\tau_R = \frac{\xi N \langle R^2 \rangle}{3\pi^2 k_B T}$$

where ξ is the monomer friction coefficient. The end-to-end vector relaxation time of the Rouse chain is then[8] $\simeq 0.8\tau_{Rouse}$. In Table S1, the parameters of the equivalent Rouse chain are estimated from the coarse-grained simulations for a low value of the dihedral barrier, $\varepsilon / T = 3.6$. The relaxation times τ_R estimated under the assumption of the Rouse model are further compared with the actual end-to-end distance (EE) and end-to-end vector (vec) relaxation times. Average dihedral relaxation times (dih) are also reported. Importantly, the end-to-end vector relaxation time is close to its Rouse time, while the end-to-to end distance relaxation time is about 3 times shorter than the end-to-end vector relaxation time, consistent with the behavior expected for a Rouse chain[9].

Table S1

**Peptide**	$<R^2>$	l_k	n_k	t_R	t_{EE}	t_{dih}	$3t_{EE}$	t_{vec}	t_{vec}/t_R
10-mer	20.25	2	5	13.7	5.3	14.5	15.9	15.2	1.1
20-mer	56.25	2.8	7	76.1	14.5	18.4	43.5	95.0	1.2
30-mer	90.25	3	10	183.1	77.6	19.3	232.8	220.0	1.2
40-mer	132.25	3.3	12	357.7	83.2	19.2	249.6	425.0	1.2

Correlations in the dihedral transitions. To find out, directly, whether transitions involving different dihedral angles are correlated, we have studied the statistics of dihedral angle flips. To this end, we mapped the time dependences of each dihedral angle onto a 3-state model (see Figure **S4** for an example). States I, II, and III correspond, respectively, to the dihedral angles in the ranges [0,120), [120,180] (or [-180,-120]), and (-120,0]. This mapping is consistent with the symmetry of the dihedral potential.

Consider the lag time between the successive flips of a dihedral angle *i* (i.e. an event where it jumps between two distinct states as defined above). In the absence of memory effects, we expect this time to be exponentially distributed, $P_i(t) = \lambda_i e^{-\lambda_i t}$, so that the jumps of this dihedral constitute a Poisson process. Indeed, we find this to be the case, Fig. S5. Now consider a pair of dihedrals, *i* and *j*. If the flipping of each is an *independent* Poisson process, then the distribution of the time lag *t* between the flip of *i* and the subsequent flip of *j* is also exponential, $P_{ij}(t) = \left(\lambda_i + \lambda_j\right) e^{-\left(\lambda_i + \lambda_j\right)t}$. For sequence-distant dihedrals, we, indeed, find this to be the case (exemplified by the case *j*=*i*+5 in Fig. S5). For the dihedrals that are close in sequence, however, $P_{ij}(t)$ deviates from a single exponential (Fig. S5), showing a bunching effect, where the average lag time between the flips of *i* and *j* is shorter than $P_{ij}(t) = \left(\lambda_i + \lambda_j\right)^{-1}$.

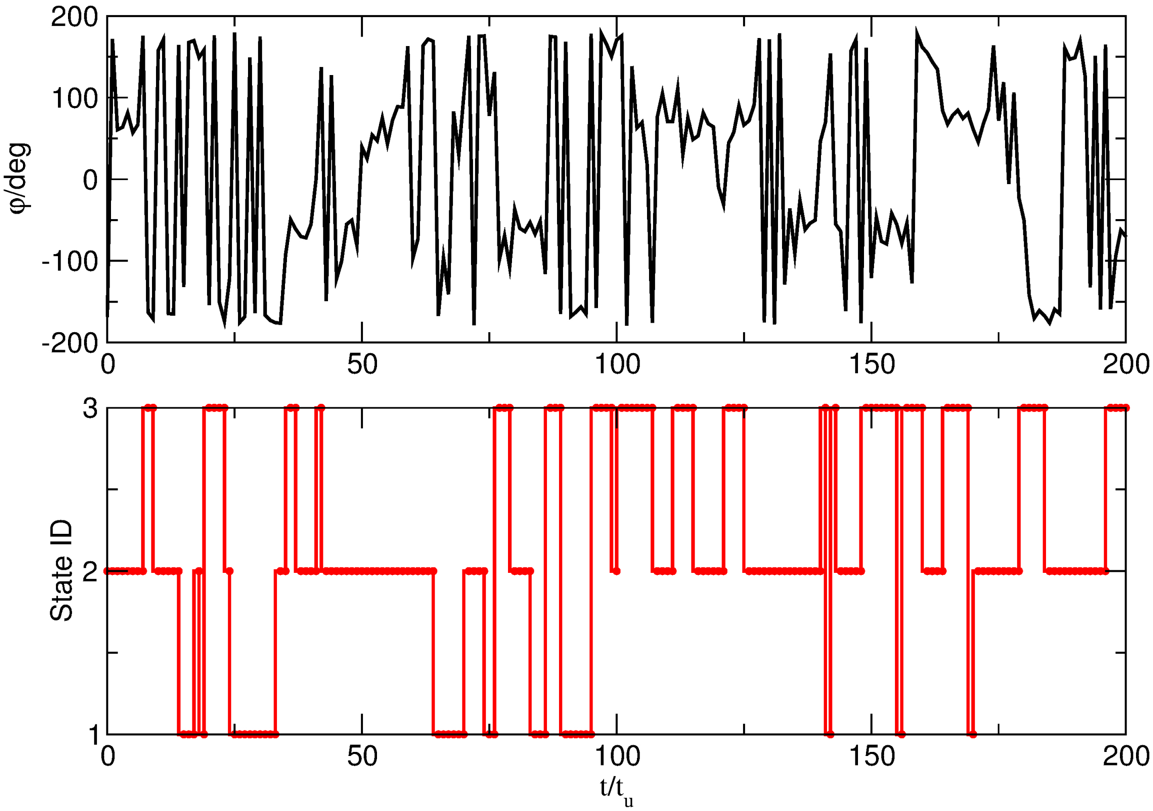

Fig. S4. Mapping of a dihedral trajectory $\varphi(t)$ onto a 3-state process.

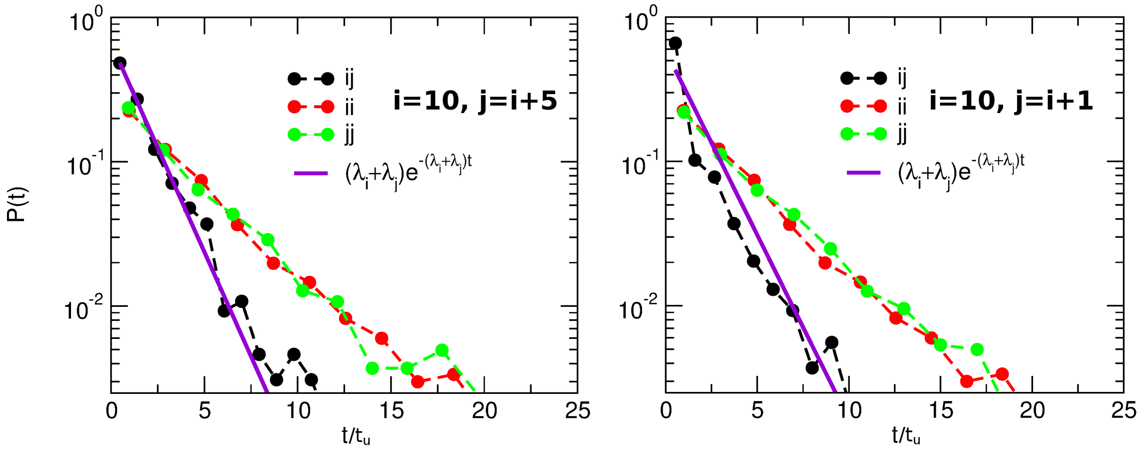

Fig. S5. Distributions of the lag time between the jumps in individual dihedrals *i* and *j* and between a jump of a dihedral *i* followed by a jump in *j* (note the logarithmic scale). The data is given for *N*=30 and $\varepsilon\ /T$= 8.

2. Simulations of the rotational isomeric state (RIS) model.

In the (alpha-carbon only, coarse grained) RIS model, the configuration of the polypeptide chain is entirely specified by its dihedral angles, $\{\varphi_1,\varphi_2,...,\varphi_{N-2}\}$, where *N* is the number of monomers. Same geometry (i.e. same bending angles) was assumed as in

Langevin dynamics of the coarse grained model described above, but the dihedrals are the only degrees of freedom in the RIS model. Each dihedral was assumed to undergo jumps between three equivalent states 1,2,3, with the same value of the jumping rate coefficients, $k_{12} = k_{21} = k_{23} = k_{32} = k_{13} = k_{31} = k$. The time evolution of each dihedral was computed using the standard kinetic Monte Carlo scheme, and the resulting trajectory of the end-to-end distance $R(t)$ was computed. The relaxation time of the end-to-end distance was computed, for different values of the chain length, as

$$\tau_{EE} = \int_0^\infty \frac{\langle R(t)R(0)\rangle - \langle R\rangle^2}{\langle R^2\rangle - \langle R\rangle^2} dt$$

As shown in Fig. S6, this time was found to be inversely proportional to the chain length.

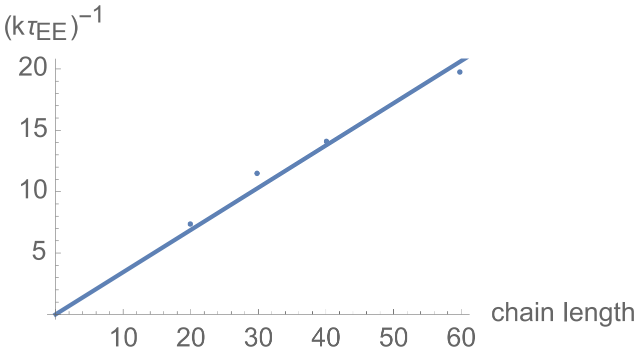

Fig. S6. Chain length dependence of the end-to-end distance relaxation time in RIS simulations, assuming that all dihedrals can rotate independently.

3. Atomistic simulations of CSP fragments

Simulation Protocol. Molecular dynamics simulations were performed using the GROMACS software package, version 4.5.5.[10] Parameters were taken from the Amber03 parameter set[11]. An extended simple point charge (SPC/E) explicit water model was used to represent the solvent. Starting from the NMR structure of the 66-residue Thermotoga maritima CSP (pdb access code 1G6P)[12], the initial peptide models were built by cutting the protein in six equal 11-residue peptide fragments. We also modeled one 11-residue peptide fragment with Gly-Ser repeat. Each of these peptides was then solvated separately in a cubic box of water molecules. The dimensions of the box were selected such that all the atoms were at all events 10 Å from any cubic wall. Counterions were added using the genion module of GROMACS, which randomly replaces water molecules with counterions in favorable locations determined by computing the electrostatic potential at the insertion site. Each system was energy minimized by, first, the steepest descent algorithm and then, by a conjugate gradient algorithm, to arrive at a conformation with no steric clashes. Each of these minimized conformations was equilibrated in two steps, with position restraints applied to all the heavy atoms throughout. The first phase involved simulating each system for 500 ps under a constant volume (NVT) ensemble. All the atoms were coupled to a bath, with the temperature kept at 300 K using the Berendsen weak coupling method. Following NVT equilibration, 500 ps of constant pressure (NPT) equilibration was accomplished to maintain pressure

isotropically at 0.138 atm. All the simulations were performed in a cubic cell employing periodic boundary conditions with the standard minimum image convention in all three directions. Long-range electrostatics was treated with the particle mesh Ewald method.[13] The cutoff used for Lennard-Jones interactions was 9 Å. Particle mesh Ewald method with a real space cut-off at 9 Å was used to account for the electrostatic interactions. All bond lenghts and all angles involving hydrogens were constrained using the LINCS algorithm.[14] An integration time step of 2 fs was used for all the simulations. Production MD runs were performed at T=300 K and P=0.138 atm in the absence of any restraints; these conditions are close to those employed in previous experimental[15] and theoretical[16] studies. The modified Berendsen thermostat[17] was used to maintain temperature, and the Parrinello-Rahman barostat[18] was used to isotropically regulate pressure during the production runs. The total length of a production run for each of the peptides was 2 μs, resulting in a total of 16 μs of simulation time.

Modified Dihedral Potentials. To explore the connection between internal friction and the dihedral energy landscape, we repeated our simulations for the 11-residue Gly-Ser repeat peptide using a softer dihedral potential, with all dihedral barriers reduced by a factor of 2, i.e., $V_n \rightarrow V_n/2$. The potential energy function describing the backbone dihedral angles has the form:

$$V(\theta) = \frac{V_n}{2}[1 + \cos(n\theta - \gamma)] \qquad (1)$$

where θ is the dihedral angle (either ϕ or ψ) and V_n is the corresponding force constant. The phase angle γ takes values of either 0° or 180°, and n is an integer that determines the periodicity of the potential.[11]

Autocorrelation Functions. We investigated the conformational dynamics of the peptides by probing the relaxation kinetics of different structural properties. Given the time dependence of a dihedral angle, $\theta(t)$, the corresponding autocorrelation function (ACF), as a function of the lag time τ, was defined as[19]:

$$C(\tau) = \langle \cos[\theta(t) - \theta(t+\tau)] \rangle \qquad (2)$$

For the end-to-end vector **R**, the ACF was defined as $C(\tau) = \langle \mathbf{R}(t)\mathbf{R}(t+\tau) \rangle$, and for the end-to-end distance R we have $C(\tau) = \langle R(t)R(t+\tau) \rangle$. The relaxation time associated with each of these parameters was computed from the normalized ACF,

$$C_N(\tau) = \frac{C(t) - C(\infty)}{C(0) - C(\infty)} \qquad (3)$$

as

$$\tau_r = \int_0^\infty C_N(t)dt \qquad (4)$$

Because of the noise in the raw ACFs, the integral of Eq. 4 was evaluated using analytic fits of $C_N(t)$. Single exponential, bi-exponential, and stretched exponential fits were, respectively, used for the end-to-end vector, dihedral angle, and end-to-end distance ACFs. Those fits along with the raw autocorrelation functions are shown in Figs. S7-S24, S33-S40.

Estimating translational diffusion coefficients. The translational diffusion coefficient of a peptide, D, was estimated from the time-dependence of its center-of-mas position $\mathbf{r}$ as

$$D = \frac{1}{6}\frac{d}{dt}\left\langle \left[\mathbf{r}(t) - \mathbf{r}(0)\right]^2 \right\rangle \quad (5)$$

As shown in Figs. S25-S32, the mean square distance $\left\langle \left[\mathbf{r}(t) - \mathbf{r}(0)\right]^2 \right\rangle$ is, indeed, a linear function of time except at very short times (tens of picoseconds and less, where the assumption of overdamped dynamics underlying Eq. 5 is no longer valid).

Dihedral angle autocorrelation functions averaged over all dihedrals within the peptide and their bi-exponential fits:

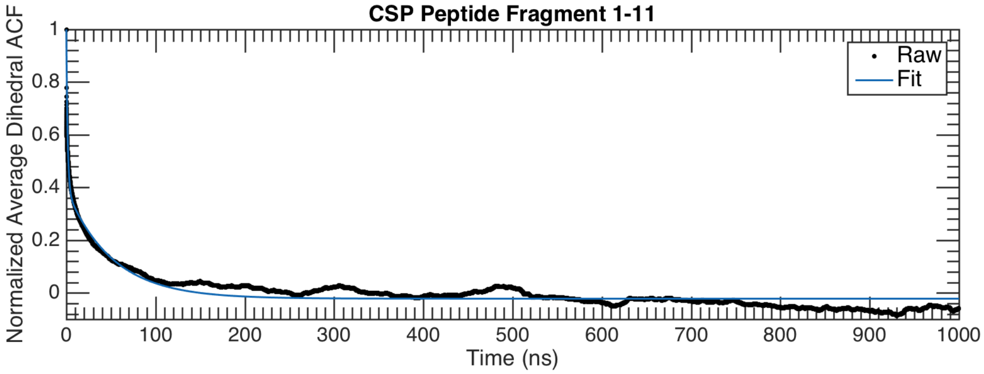

Fig. S7

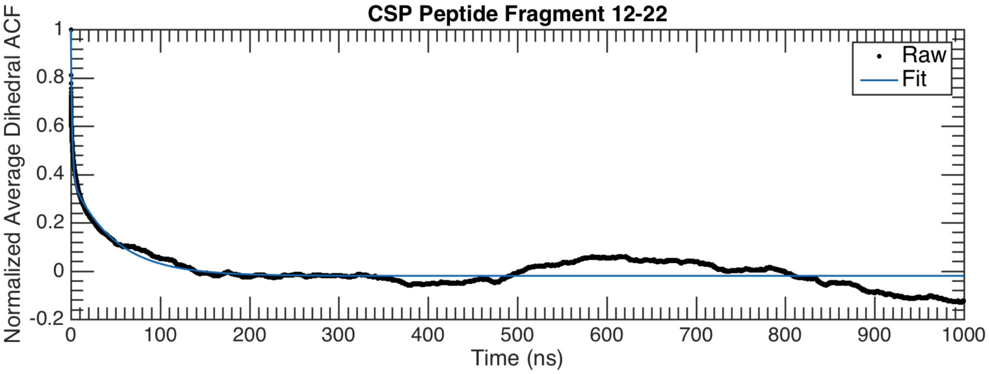

Fig. S8

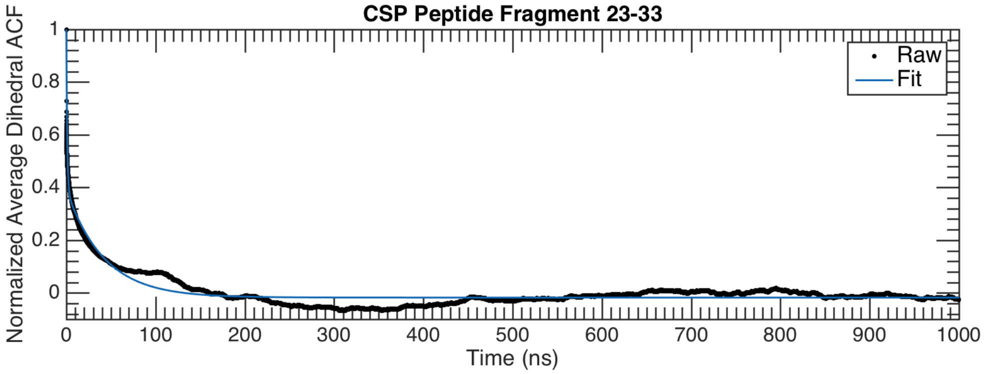

Fig. S9

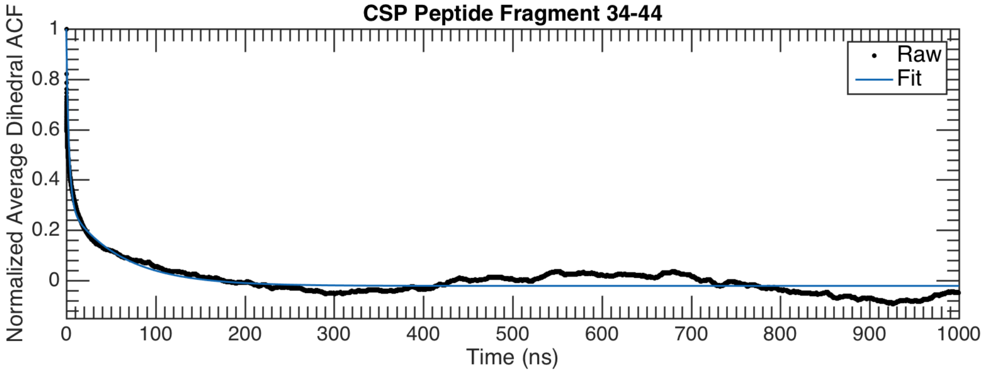

Fig. S10

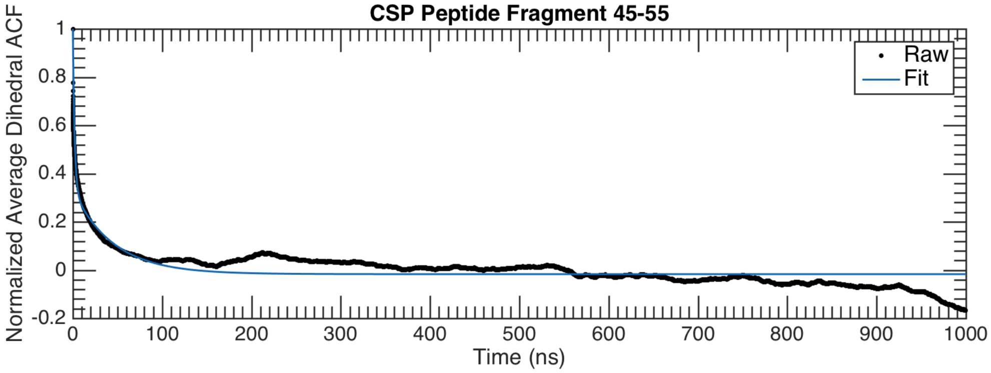

Fig. S11

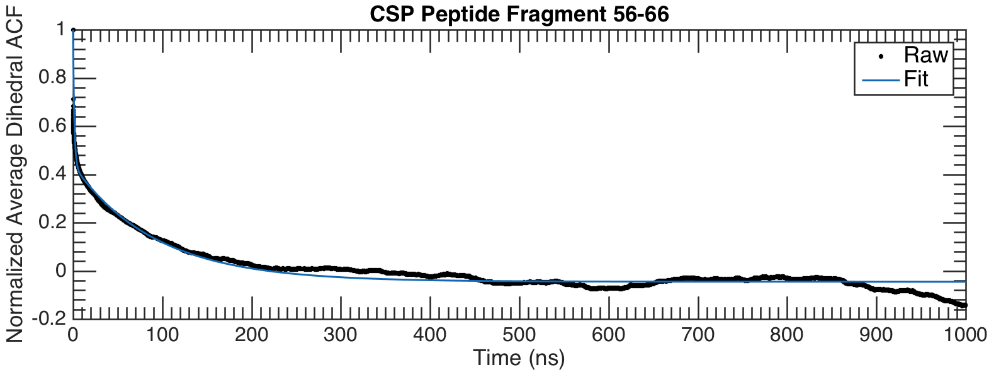

Fig. S12

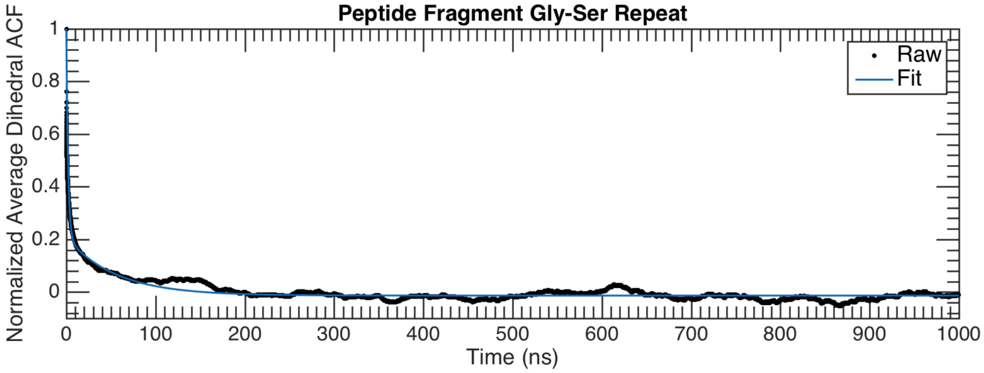

Fig. S13

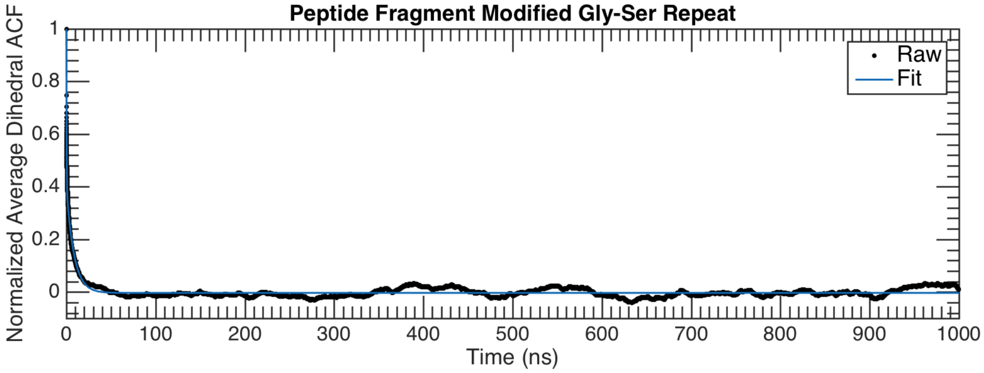

Fig. S14

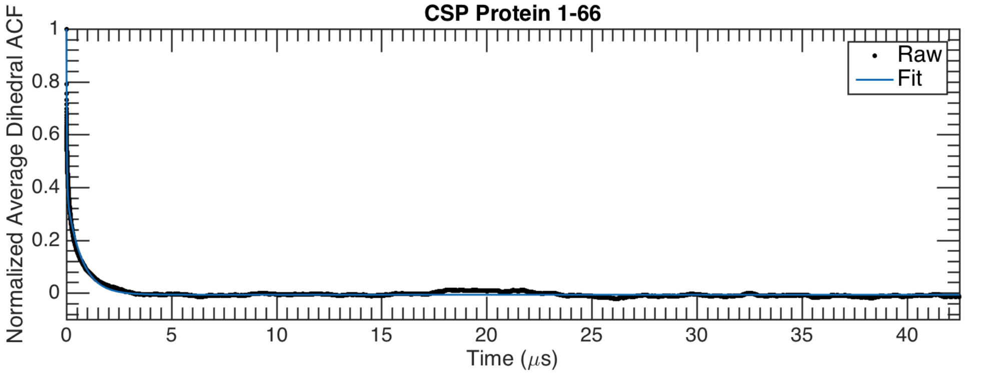

Fig. S15

End-to-end distance autocorrelation functions and their stretched-exponential fits:

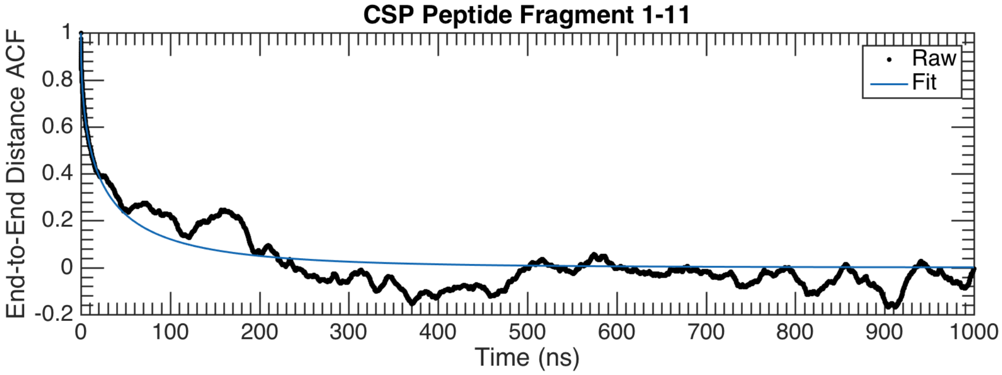

Fig. S16

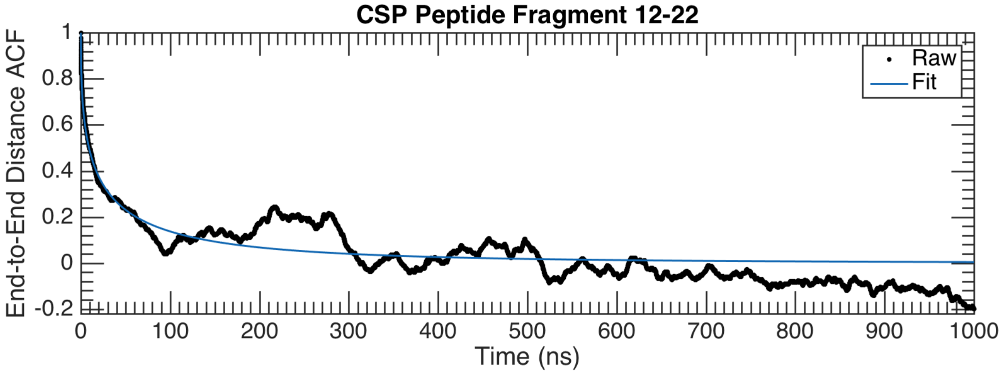

Fig. S17

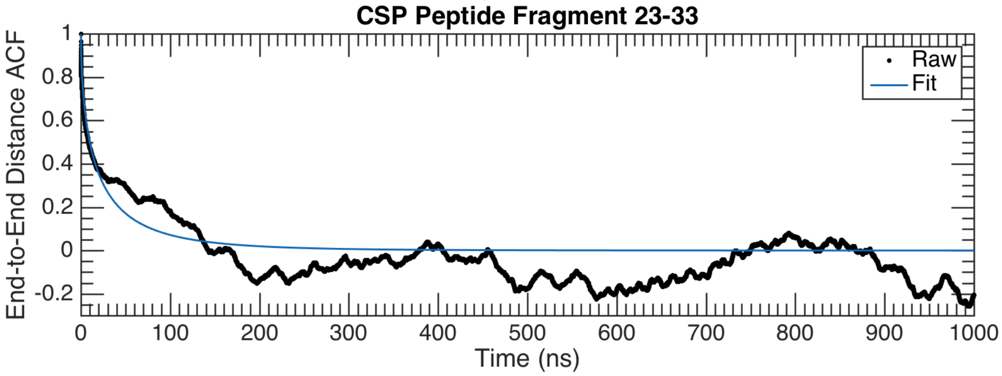

Fig. S18

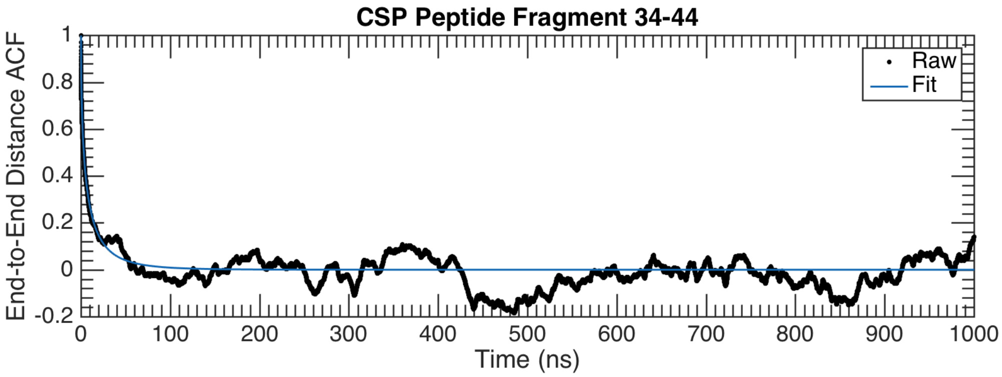

Fig. S19

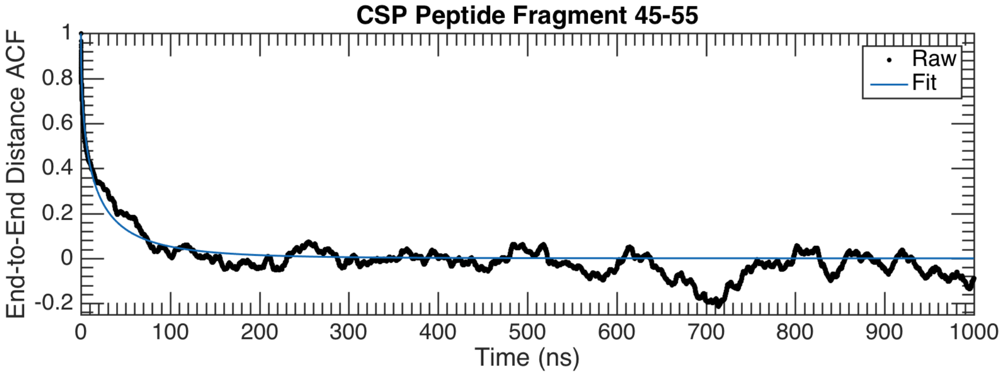

Fig. S20

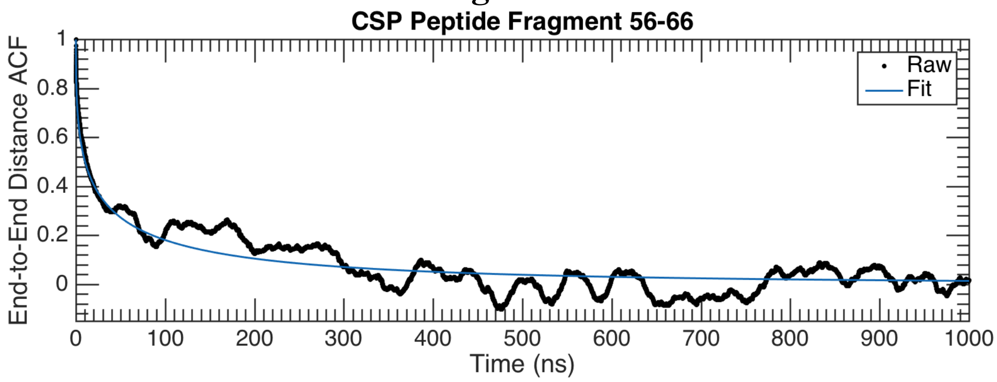

Fig. S21

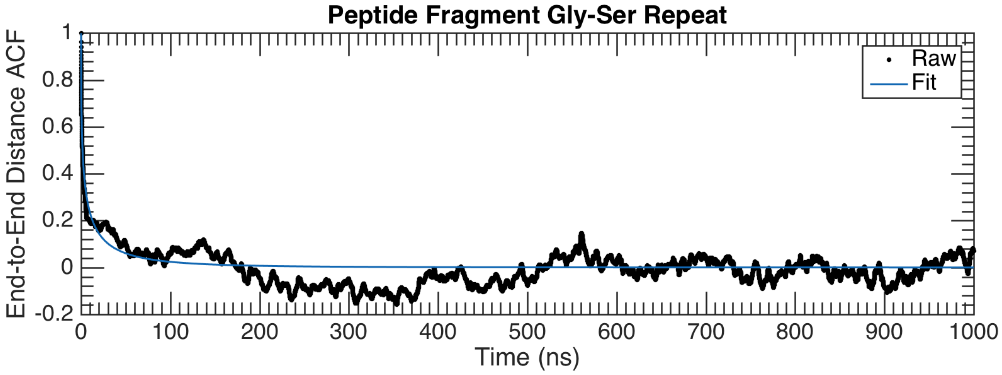

Fig. S22

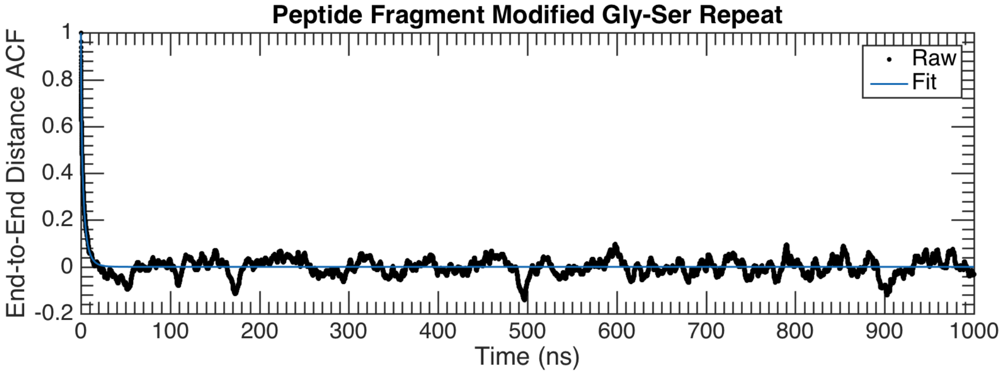

Fig. S23

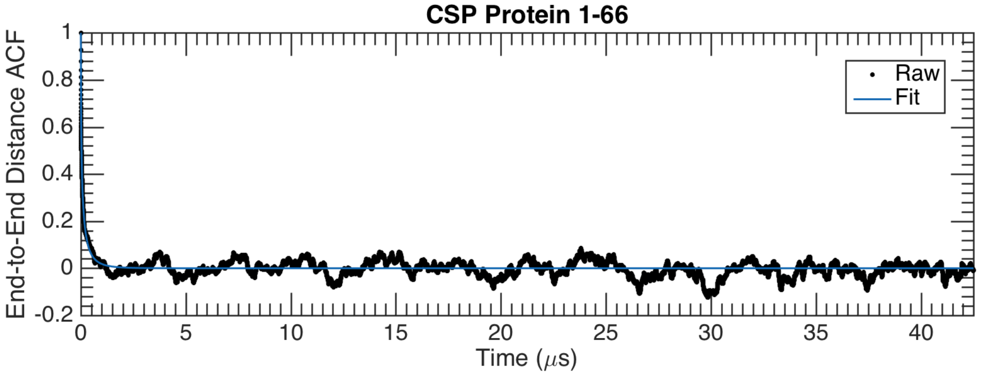

Fig. S24

Mean square displacement vs. time profiles:

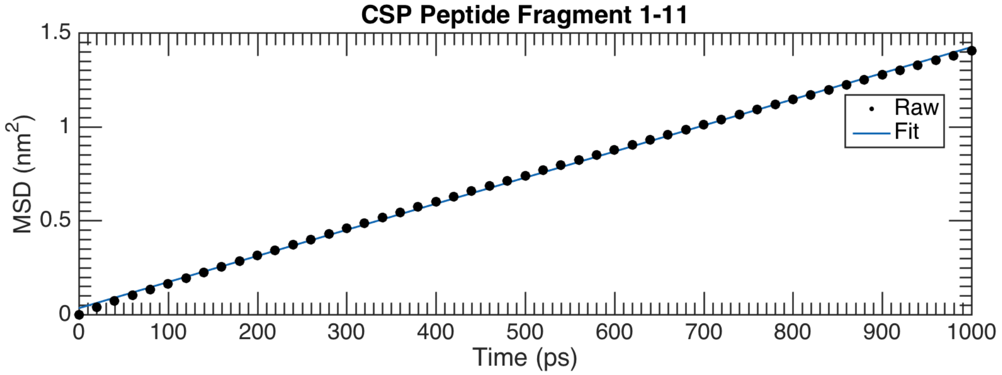

Fig. S25

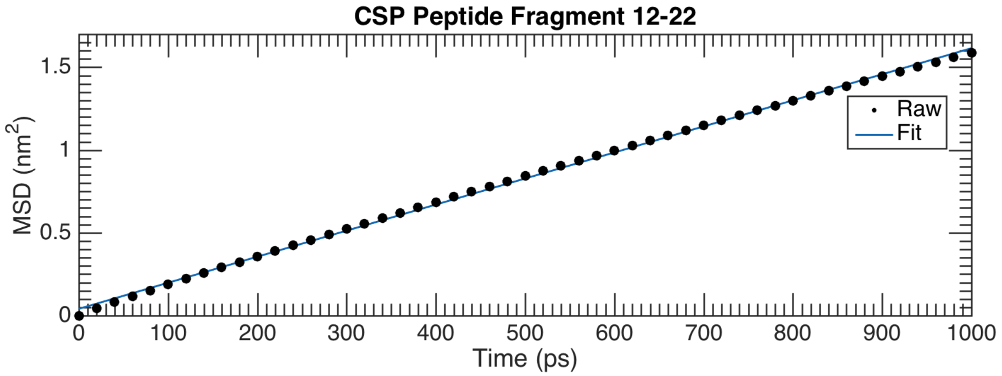

Fig. S26

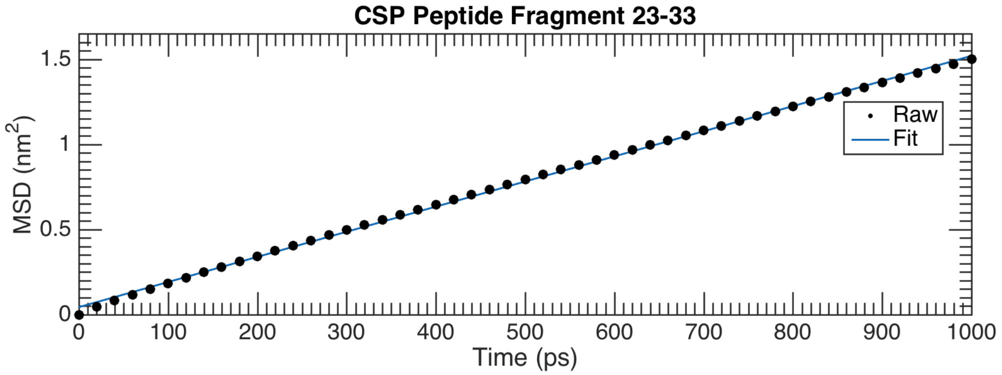

Fig. S27

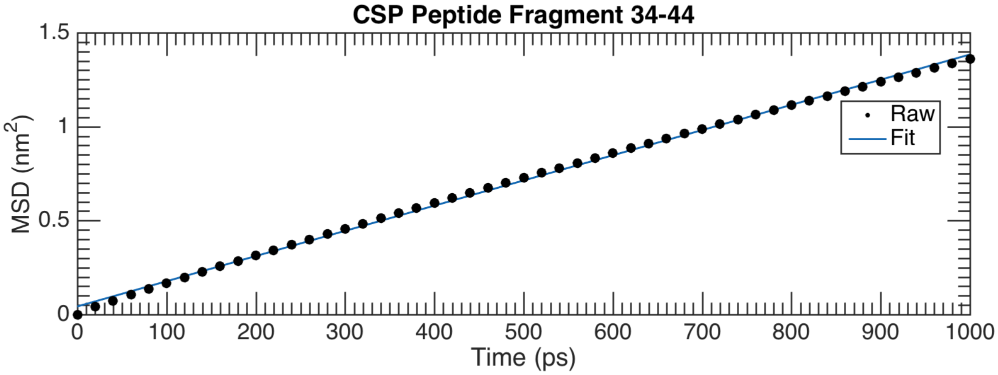

Fig. S28

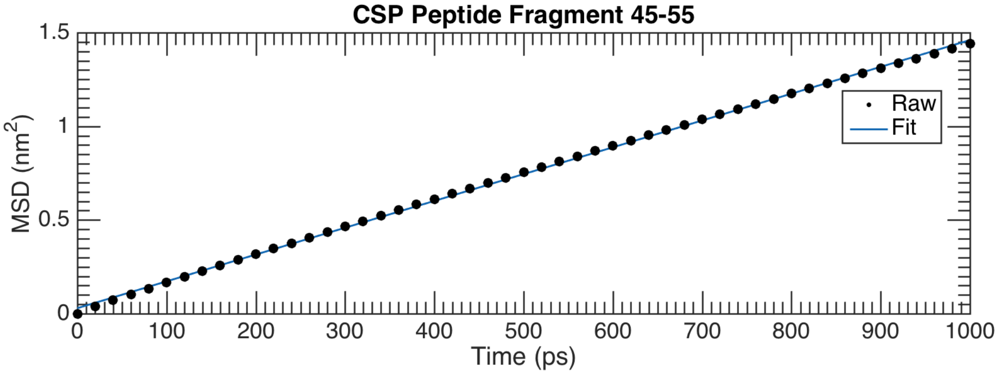

Fig. S29

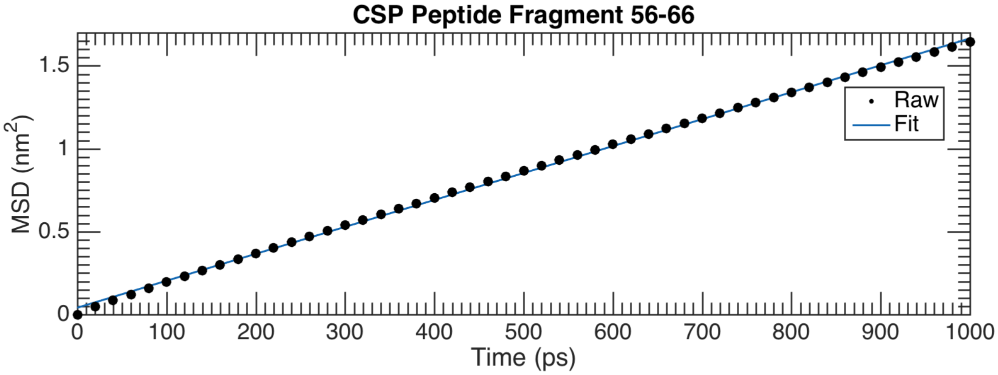

Fig. S30

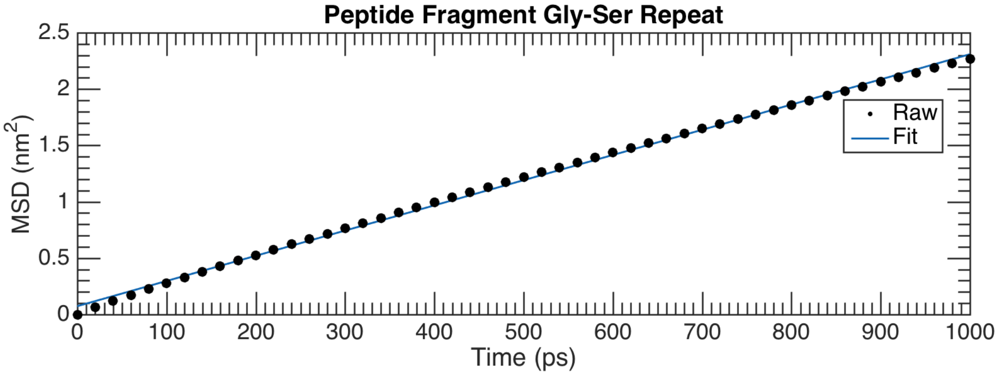

Fig. S31

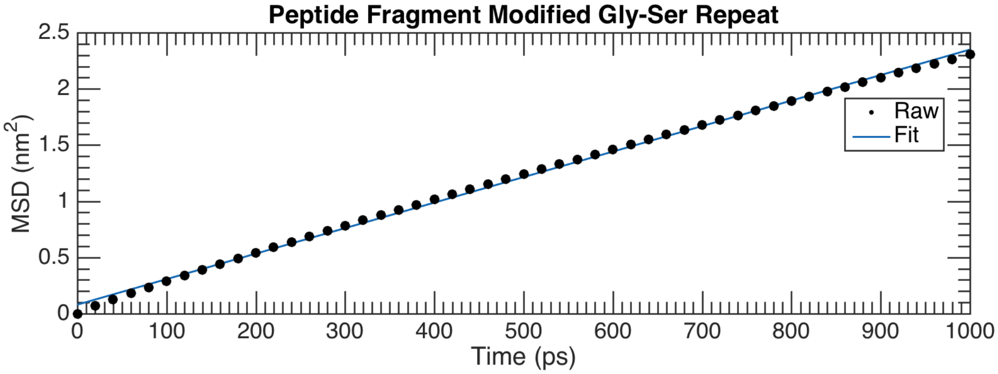

Fig. S32

End-to-end vector autocorrelation functions and their exponential fits:

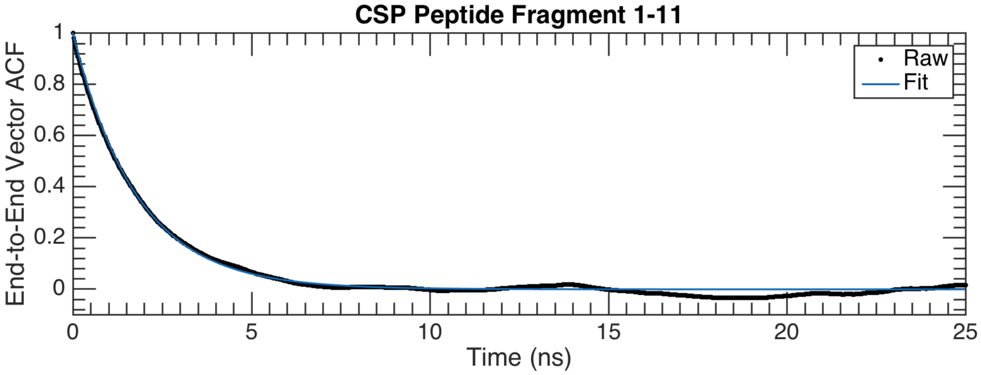

Fig. S33

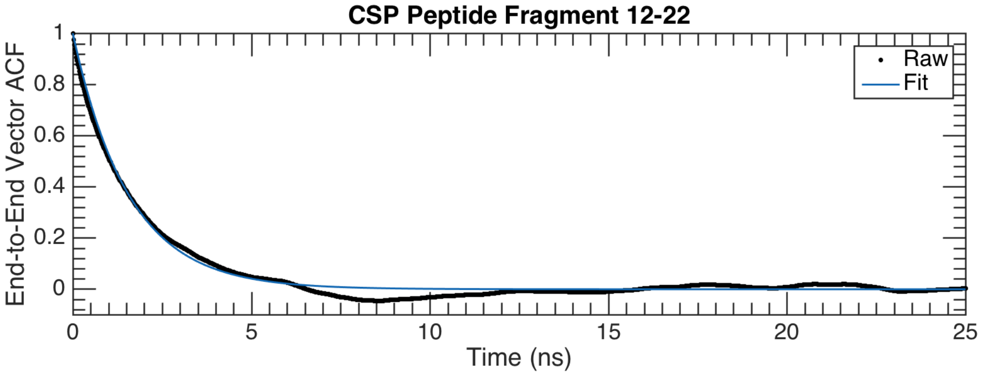

Fig. S34

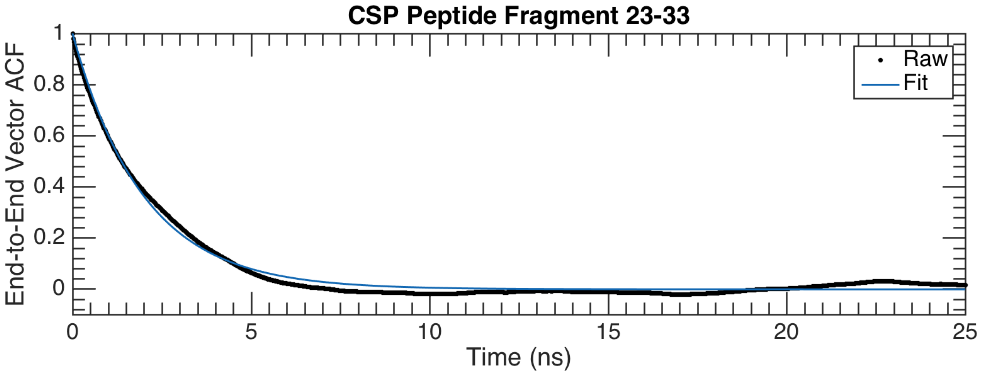

Fig. S35

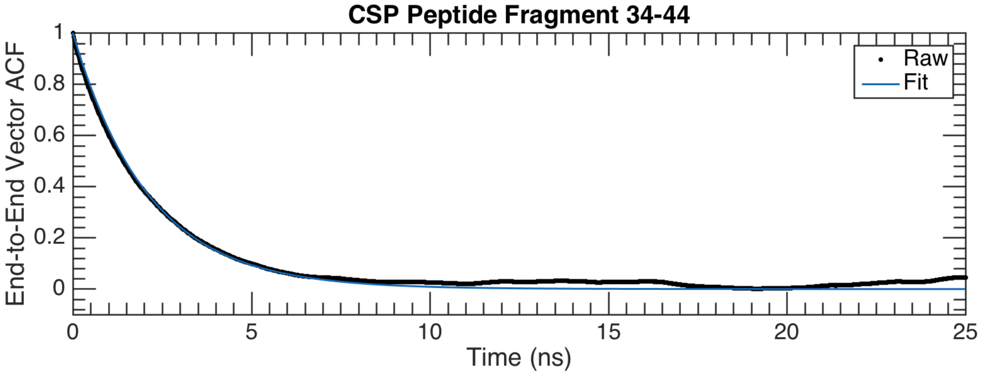

Fig. S36

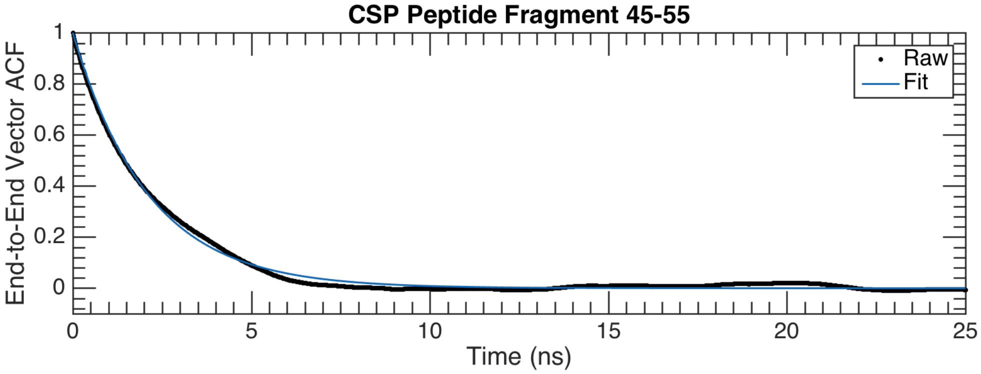

Fig. S37

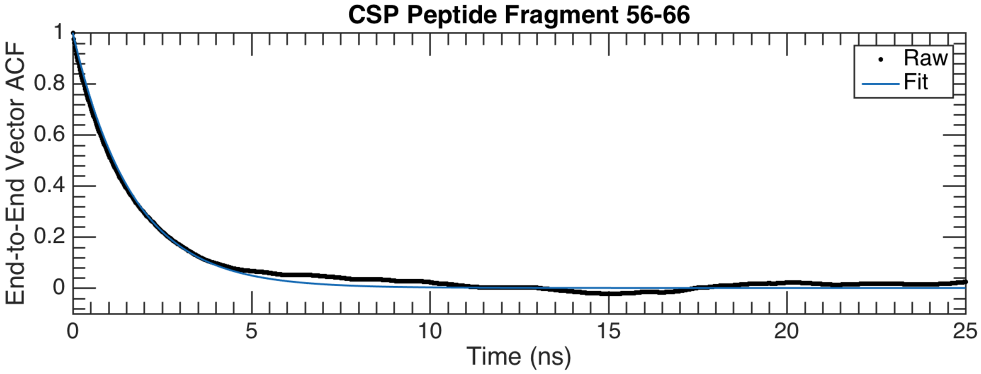

Fig. S38

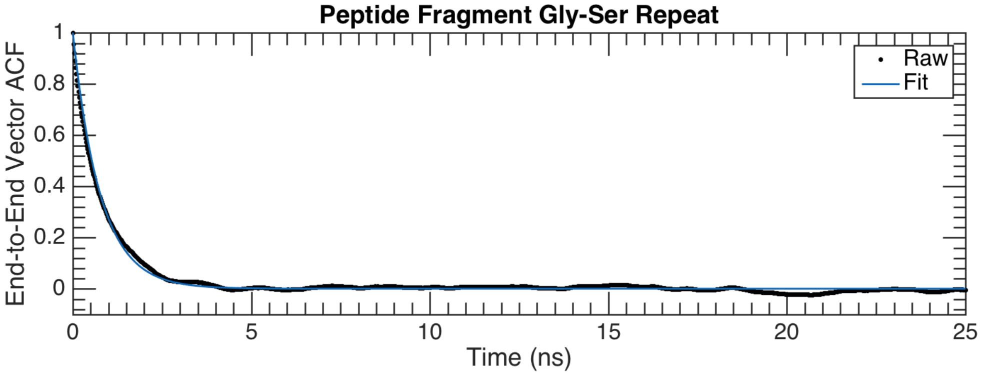

Fig. S39

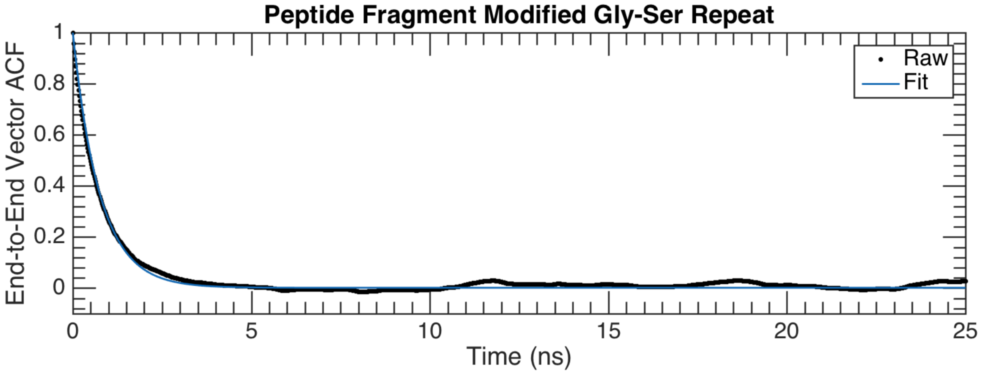

Fig. S40

Decay times of individual dihedral angle ACFs vary over many orders of magnitudes. Here we show two representative examples:

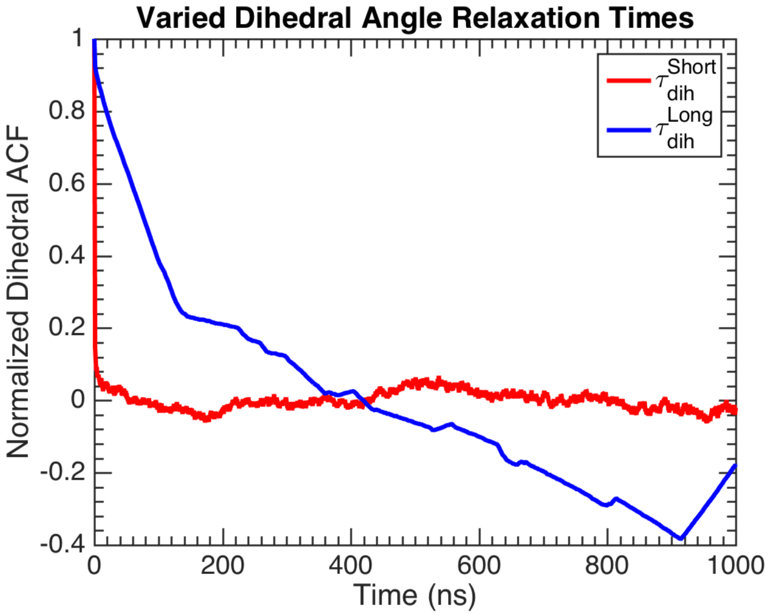

Fig. S41

Reference:

1. Honeycutt, J.D. & Thirumalai, D. The nature of folded states of globular-proteins. *Biopolymers* **32**, 695-709 (1992).

2. Makarov, D.E. Spatiotemporal correlations in denatured proteins: The dependence of fluorescence resonance energy transfer (FRET)-derived protein reconfiguration times on the location of the FRET probes. *J. Chem. Phys.* **132**, 035104 (2010).

3. Sorenson, J.M. & Head-Gordon, T. Towards minimalist models of larger proteins: A ubiquitin-like protein. *PROTEINS: Struct. Funct. Genet.* **46**, 368-379 (2002).

4. Sorenson, J.M. & Head-Gordon, T. Protein engineering tudy of protein L by simulation. *J. Comput. Biol.* **9**, 35-54 (2002).

5. Veitshans, T., Klimov, D. & Thirumalai, D. Protein folding kinetics: Timescales, pathways and energy landscapes in terms of sequence-dependent properties. *Fold. Design* **2**, 1 (1996).

6. Huang, L., Kirmizialtin, S. & Makarov, D.E. Computer simulations of the translocation and unfolding of a protein pulled mechanically through a pore. *The J. Chem. Phys.* **123**, 124903 (2005).

7. Huang, L. & Makarov, D.E. Translocation of a knotted polypeptide through a pore. *J. Chem. Phys.* **129**, 121107 (2008).

8. Cheng, R.R., Hawk, A.T. & Makarov, D.E. Exploring the role of internal friction in the dynamics of unfolded proteins using simple polymer models. *J. Chem. Phys.* **138**, 074112 (2013).

9. Portman, J.J. Non-Gaussian dynamics from a simulation of a short peptide: Loop closure rates and effective diffusion coefficients. *J. Chem. Phys.* **118**, 2381-2391 (2003).

10. Pronk, S. et al. GROMACS 4.5: a high-throughput and highly parallel open source molecular simulation toolkit. *Bioinformatics* **29**, 845-854 (2013).

11. Duan, Y. et al. A point-charge force field for molecular mechanics simulations of proteins based on condensed-phase quantum mechanical calculations. *J. Comput. Chem.* **24**, 1999-2012 (2003).

12. Kremer, W. et al. Solution NMR structure of the cold-shock protein from the hyperthermophilic bacterium Thermotoga maritima. *Eur. J. Biochem.* **268**, 2527-2539 (2001).

13. Darden, T., York, D. & Pedersen, L. Particle mesh Ewald: An N · log (N) method for Ewald sums in large systems. *J. Chem. Phys.* **98**, 10089-10092 (1993).

14. Hess, B. P-LINCS: A parallel linear constraint solver for molecular simulation. *J. Chem. Theory Comput.* **4**, 116-122 (2008).

15. Soranno, A. et al. Quantifying internal friction in unfolded and intrinsically disordered proteins with single-molecule spectroscopy. *Proc. Natl. Acad. Sci. USA* **109**, 17800-17806 (2012).

16. Echeverria, I., Makarov, D.E. & Papoian, G.A. Concerted dihedral rotations give rise to internal friction in unfolded proteins. *J. Am. Chem. Soc.* **136**, 8708-8713 (2014).

17. Bussi, G., Donadio, D. & Parrinello, M. Canonical sampling through velocity rescaling. *J. Chem. Phys.* **126**, 014101 (2007).

18. Parrinello, M. & Rahman, A. Polymorphic transitions in single crystals: A new molecular dynamics method. *J. Appl. Phys.* **52**, 7182-7190 (1981).

19. van der Spoel, D. & Berendsen, H. Molecular dynamics simulations of Leu-enkephalin in water and DMSO. *Biophys. J.* **72**, 2032 (1997).
